# Benchmarking of protein descriptor sets in proteochemometric modeling (part 2): modeling performance of 13 amino acid descriptor sets

**DOI:** 10.1186/1758-2946-5-42

**Published:** 2013-09-24

**Authors:** Gerard JP van Westen, Remco F Swier, Isidro Cortes-Ciriano, Jörg K Wegner, John P Overington, Adriaan P IJzerman, Herman WT van Vlijmen, Andreas Bender

**Affiliations:** 1 Division of Medicinal Chemistry, Leiden / Amsterdam Center for Drug Research, Einsteinweg 55, Leiden 2333, CC, The Netherlands; 2 Structural Biology and Chemistry Department, Unité de Bioinformatique Structurale, Institut Pasteur and CNRS URA 2185, 25-28, rue du Dr. Roux, Paris 75 724, France; 3 Tibotec BVBA, Turnhoutseweg 30, Beerse 2340, Belgium; 4 ChEMBL Group, European Molecular Biology Laboratory European Bioinformatics Institute (EMBL-EBI), Wellcome Trust Genome Campus, Hinxton CB10 1SD, United Kingdom; 5 Department of Chemistry, Unilever Centre for Molecular Science Informatics, University of Cambridge, Lensfield Road, Cambridge CB2 1EW, United Kingdom

**Keywords:** GPCR, HIV, QSAM, Peptides, Amino acid index, Protein descriptors, Polypharmacology

## Abstract

**Background:**

While a large body of work exists on comparing and benchmarking descriptors of molecular structures, a similar comparison of protein descriptor sets is lacking. Hence, in the current work a total of 13 amino acid descriptor sets have been benchmarked with respect to their ability of establishing bioactivity models. The descriptor sets included in the study are Z-scales (3 variants), VHSE, T-scales, ST-scales, MS-WHIM, FASGAI, BLOSUM, a novel protein descriptor set (termed ProtFP (4 variants)), and in addition we created and benchmarked three pairs of descriptor combinations. Prediction performance was evaluated in seven structure-activity benchmarks which comprise Angiotensin Converting Enzyme (ACE) dipeptidic inhibitor data, and three proteochemometric data sets, namely (1) GPCR ligands modeled against a GPCR panel, (2) enzyme inhibitors (NNRTIs) with associated bioactivities against a set of HIV enzyme mutants, and (3) enzyme inhibitors (PIs) with associated bioactivities on a large set of HIV enzyme mutants.

**Results:**

The amino acid descriptor sets compared here show similar performance (<0.1 log units RMSE difference and <0.1 difference in MCC), while errors for individual proteins were in some cases found to be larger than those resulting from descriptor set differences ( > 0.3 log units RMSE difference and >0.7 difference in MCC). Combining different descriptor sets generally leads to better modeling performance than utilizing individual sets. The best performers were Z-scales (3) combined with ProtFP (Feature), or Z-Scales (3) combined with an average Z-Scale value for each target, while ProtFP (PCA8), ST-Scales, and ProtFP (Feature) rank last.

**Conclusions:**

While amino acid descriptor sets capture different aspects of amino acids their ability to be used for bioactivity modeling is still – on average – surprisingly similar. Still, combining sets describing complementary information consistently leads to small but consistent improvement in modeling performance (average MCC 0.01 better, average RMSE 0.01 log units lower). Finally, performance differences exist between the targets compared thereby underlining that choosing an appropriate descriptor set is of fundamental for bioactivity modeling, both from the ligand- as well as the protein side.

## Background

### Proteochemometric modeling

Proteochemometric (PCM) modeling uses statistical modeling techniques to model the ligand–target interaction space [1-4]. The technique is similar to Quantitative Structure-Activity Relationship (QSAR) modeling but expands on its ligand-only nature in that it takes *both* ligand- and target space into account when generating bioactivity models. This enables PCM to explain bioactivity based on chemical properties (features of the ligand) in combination with particular protein properties (features of the target). Moreover, PCM models are able to extrapolate in both the chemical (ligand) as well as the biological (target) domain (under the limitations of the data and the models constructed), as shown in previous work [5-7]. Given that both ligand- and target descriptors are used for PCM models, it follows that the target description is as important as the ligand description. While several publications are available benchmarking ligand descriptors [8-10], on the side of target descriptor sets there is significantly less literature currently available. Generally peptide descriptor sets obtained from the field of Quantitative Sequence-Activity Modeling (QSAM) are used in PCM [1,11-15]. However descriptors taking three-dimensional information into account have also been used in previous studies [16-20]. Still, these descriptors require structural information, which is not always available. In order to have a method at hand that is applicable as widely as possible the performance of sequence-based descriptors is compared in the current work. For a further rationale of the current work the reader is referred to the companion paper [21].

### Amino acid descriptor sets considered in this study

In the current work a total of 13 different individual descriptor sets have been benchmarked which belong to descriptor classes that are derived in conceptually different ways (Table 1; descriptor set names are consistent with our previous study) [21]. Firstly, three descriptor sets, namely Z-scales (3 PCs, 5 PCs, or Binned) [6,7,14], VHSE [22], and ProtFP PCA (3 PCs, 5 PCs, or 8 PCs), are based on a PCA analysis of physicochemical properties. Secondly, ST-Scales and T-Scales consist of a principal component analysis of mostly topological properties [23,24]. FASGAI, part of the third category of descriptor sets tested, is based on a factor analysis of physicochemical properties [25]. Furthermore, two descriptor sets were tested that are calculated in a very different manner compared to the first six, namely a descriptor set based on three dimensional electrostatic properties calculated per AA (MS-WHIM) [26]. Additionally, a descriptor set based on a VARIMAX analysis of physicochemical properties which were subsequently converted to indices based on the BLOSUM62 substitution matrix (BLOSUM) [27].Furthermore a descriptor set only describing each AA by a single feature was tested ProtFP (Feature) [5,28]. Additionally three different combinations of descriptor sets also sampled individually were benchmarked. The paired sets were: ProtFP (Feature) and Z-Scales (3), ProtFP (PCA3) and Z-Scales (Binned). The rationale for these two combinations was that the information should be complementary and this would lead to better performance. Finally, Z-Scales (3) was also combined with an average value and standard deviation of all Z-scales for the amino acids of the target in question; this was called Z-Scales (3) and Z-Scales (Avg). The rationale here was that adding an average value and standard deviation of for example Z1 would provide an average lipophilicity value for a binding pocket (in case of the GPCRs for instance), which could add information. Please see Table 1 and the first, related study for details of the descriptor sets compared.

**Table 1:** Amino acid descriptor sets compared in the current study

**Descriptor set**	**Type**	**Derived by**	**# of components**	**Variance explained**	**AAs covered**
BLOSUM	Physicochemical and substitution matrix	VARIMAX	10	n/a	20
FASGAI	Physicochemical	Factor Analysis	6	84%	20
MSWHIM	3D electrostatic potential	PCA	3	61%	20
ProtFP (PCA3)	Physicochemical	PCA	3	75%	20
ProtFP (PCA5)	Physicochemical	PCA	5	83%	20
ProtFP (PCA8)	Physicochemical	PCA	8	92%	20
ProtFP (Feature)	Feature based	Hashing	n/a	n/a	20
ST-scales	Topological	PCA	5	91%	167
T-scales	Topological	PCA	8	72%	135
VHSE	Physicochemical	PCA	8	77%	20
Z-scales (3)	Physicochemical	PCA	3	n/a	87
Z-scales (5)	Physicochemical	PCA	5	87%	87
Z-scales (Binned)	Physicochemical	PCA followed by binning	n/a	n/a	20
ProtFP (Feature) and Z-Scales (3)	Physicochemical and Feature Based	PCA and Hashing	n/a	n/a	20
Z-Scales (3) and Z-Scales (Avg)	Physicochemical	PCA and target average	n/a	n/a	20
ProtFP (PCA3) and Z-Scales (Binned)	Physicochemical	PCA and binning	n/a	n/a	20

The first column contains the name of the descriptor set as used in the main text. Further listed are the type, dimensionality reduction, number of components and variance of the original matrix explained. The last column differentiates between descriptor sets only covering the natural amino acids or more. Not available is abbreviated by n/a.

### Summary of the benchmarking performed

In the current work all descriptor sets are used on four different data sets by constructing structure-bioactivity models and comparing their performance (see Table 2 for details). The datasets are firstly a previously published set of 58 dipeptides that have an inhibitory effect on the angiotensin-converting enzyme (ACE) [29]; secondly, a set of 32 GPCRs and approximately 100 active and 100 inactive compounds per receptor obtained from ChEMBL-16 [30]; a set of 451 non-nucleoside reverse transcriptase inhibitors (NNRTIs) tested for activity against 14 HIV mutants (used in a previous publication where the protein descriptor set was kept constant throughout the study) [28]; and finally a set of 9 clinically approved protease inhibitors (PIs) tested for activity against a panel of 1060 HIV mutants (used in a previous publication where the protein descriptor was kept constant) [7].

**Table 2:** Data sets used for the bioactivity benchmarks

	**ACE inhibitors**	**GPCRs**	**NNRTIs**	**PIs**
Total size (data points)	58	6,046	4,024	6,995
Total compounds	n/a	3,230	451	9
Average compound tanimoto distance (ECFP_6)	n/a	0.92	0.54	0.73
Average euclidian distance compounds (physicochemical)	n/a	1.28	n/a	0.90
Total targets (peptides / proteins)	58	32	14	1060
Average target tanimoto distance (ProtFP (Feature))	0.83	0.22	0.14	0.03
Average euclidian distance target (ProtFP (PCA3))	1.35	0.93	0.44	0.26
Completeness (% of total compound - target pairs)	n/a	0.06	0.64	0.73

The datasets were selected to obtain a diverse collection of sets amenable to PCM modeling. To this extend both GPCRs and enzymes were included. Moreover sets representing initial hit discovery, lead optimization, and well-established structure-activity space modeling were included.

### Aim of the work

As outlined in the companion paper, several of the descriptor sets compared here are derived from a large number of (up to 147) non-natural amino acids, leading to the hypothesis that for natural amino acid their resolution might not be optimal [21]. Additionally, it was observed that using more PCs from a descriptor set per amino acid residue does change descriptor behavior while adding typically less information than in the first two or three PCs [21].

Building on the results from the other paper, the main aim of the current work is twofold. Firstly, it stands to reason if one should use all PCs form a descriptor set or not (and to quantify the value there is to gain from using more or less PCs). Secondly, the aim was to identify the optimal descriptor for use in PCM based models (and if one can better use a descriptor focused on natural amino acids or not).

## Results and discussion

### 70–30 validation on ACE inhibitors

**Figure 1:**
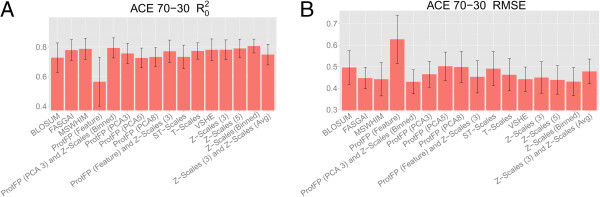
**Mean performance of the benchmarked descriptor sets in the ACE inhibitors 70–30 validation experiments.** The mean is calculated over ten different experiments and the error bars represent the standard deviation. Shown are the R_0_^2^**(A)** and the RMSE **(B)**. It can be seen that Z-scales (Binned), and ProtFP (PCA3) combined with Z-Scales (Binned) performed the best on this dataset, followed by Z-scales (5). The ProtFP (Feature) descriptor set showed worst performance in this case.

The first benchmark performed on the dataset of dipeptides inhibiting ACE was a 70–30 validation experiment where a random 70% of the data set was used for training and 30% for testing. The results of this validation on the test set are shown in Figure 1. The figure shows that all descriptor sets capture the bioactivity space of the peptides reasonably well, represented by RMSE values on the test set of below 0.700 log units. The best performing descriptor sets are Z-scales (Binned) (RMSE is 0.430 log units and the R_0_^2^ is 0.794), and ProtFP (PCA3) with Z-Scales (Binned) (RMSE 0.431 log units and R_0_^2^ 0.806), followed by Z-Scales (5) (RMSE 0.439 log units and R_0_^2^ 0.790) and MS-WHIM (RMSE 0.442 log units and R_0_^2^ 0.787). The worst performing descriptor set is ProtFP (Feature) (RMSE 0.627 log units and R_0_^2^ 0.566). Given that the ProtFP (Feature) descriptor set merely encodes for presence or absence of features (amino acids) and that dipeptides are modeled (hence only two features per datapoint) a slightly lowered performance was expected. The BLOSUM, ProtFP (PCA5) and ProtFP (PCA8) (RMSE 0.496 - 0.502 log units and R_0_^2^ 0.732 - 0.726) descriptor sets are performing better than ProtFP (Feature), but are still lagging compared to the above descriptor sets. This was not expected as these descriptor sets provide a continuous value description like the others. The numerical values for the RMSE, Q^2^ and R_0_^2^ of the model fit are included as Additional file 1: Table S1, which also includes the training parameters Q^2^ and cross validated RMSE (CV_RMSE) compared to previously published studies for the same descriptor sets on the same data set. This table shows that the current approach using random forest models performs on par or better than previously published models and hence demonstrates reproducibility of published results. In order to gain further insight into descriptor set performance, a PCA analysis was performed of the similarity space formed by the dipeptides.

### ACE inhibitor activity space

**Figure 2:**
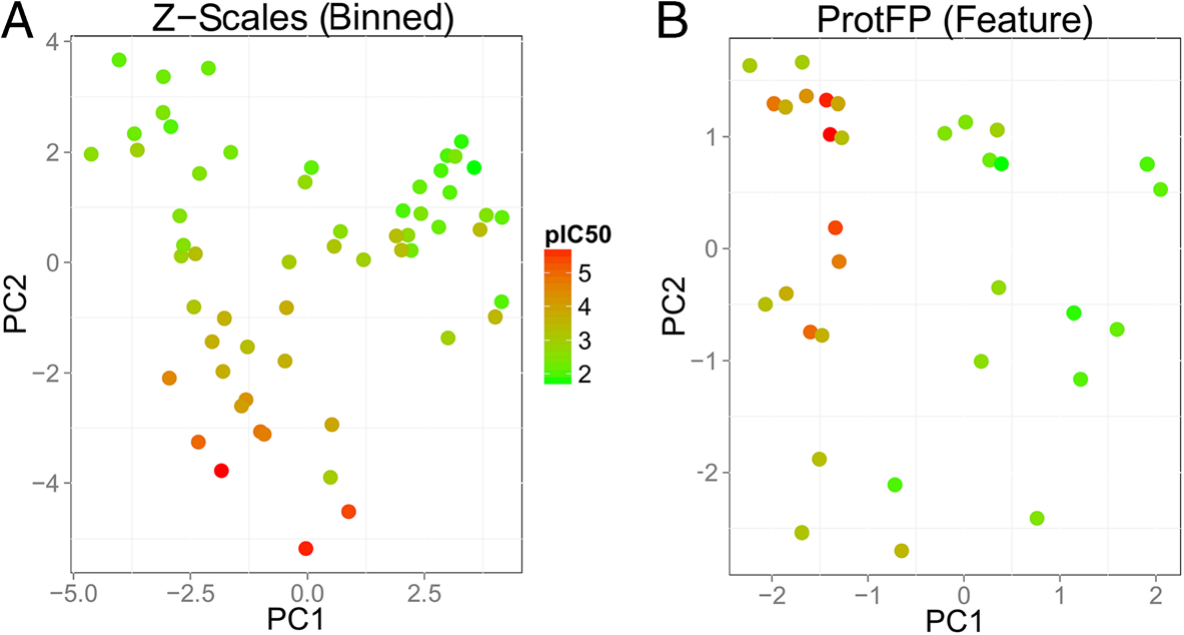
**PCA plot of ACE inhibitor similarity. (A)** Shown are the best performing descriptor set in the ACE inhibitor experiment (Z-Scales (Binned)), and **(B)** the worst performing descriptor set (ProtFP (Feature)). From the plot the reasons for their respective performance becomes apparent as part A of the figure shows a clear distribution in space correlating with the activity (indicated by the color), which is less the case for **(B)**.

The first two principal components for each set are shown in Figure 2, colored the points by their pIC_50_ values (Figure 2, and Additional file 1: Figures S1, S2, S3). The figure visually represents the degree to which descriptor sets exhibit ‘Neighbourhood Behavior’ [31]. A direct correlation is observed in the Z-scales (Binned) descriptor set between location in PCA space and activity, high-affinity peptides score negatively on PC2, whereas all marginally active compounds score 0 or higher. It can be seen that the way the descriptor set characterizes the peptides’ similarity corresponds to their bioactivity, which is in accordance with the ‘Similar Property Principle’ and in turn results in better bioactivity models. Conversely, the pattern obtained from the ProtFP (Feature) descriptor set does not clearly separate actives and inactives, explaining the poor performance of this descriptor set. The well-performing descriptor sets ProtFP (PCA3), Z-scales (3) and MS-WHIM also display a clustering similar to Z-scales (Binned). The PCA shows the highly active peptides to cluster together and the lesser actives are separated from these actives. Hence, overall investigating the ‘Neighbourhood Behavior’ in the descriptor spaces considered rationalizes why descriptor sets perform better or worse on this dataset.

### Conclusions ACE inhibitors

The random forest method allows recreation of models based on the individual descriptor sets that are comparable or better than the original publications for the descriptor sets benchmarked here. Differences in performance are rationalized by the fact that each descriptor set describes the AA space differently (as also shown in the companion paper) [21]. Still, most of the descriptor sets were able to generate reasonably well-performing models, which are explored in the context of PCM modeling in the following sections.

### 70–30 validation on GPCR ligands

**Figure 3:**
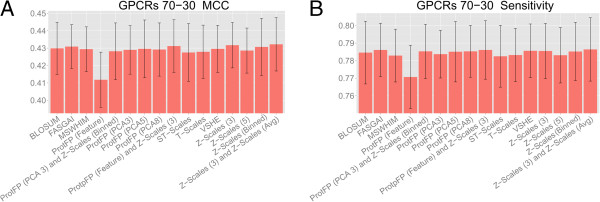
**Mean performance of the benchmarked descriptor sets in the GPCR 70–30 validation experiments.** The mean is calculated over all 32 receptors (performed 10 times) and the error bar represents the standard deviation. Shown are the MCC **(A)** and the sensitivity **(B)**. The differences between individual descriptor sets are smaller (MCC difference < 0.030, sensitivity difference < 0.020) than in the ACE inhibitor experiments, likely due to the fact that models are based on both chemical and protein similarity. For individual receptors larger performance differences occur (mean MCC difference 0.712, mean sensitivity difference 0.231) (See Additional file 1: Figure S4 for details). Z-scales (3) perform the best on this dataset, while ProtFP (Feature) performs the worst.

In a similar spirit to the validation on ACE inhibitors, a similar 70–30 validation was performed on the GPCR set. In this case a classification model was employed and performance was expressed as mean sensitivity and mean Matthews correlation coefficient (MCC) for all descriptor sets in the study (details are visualized in Figure 3, see also Methods) [32]. Here the descriptor sets perform very similar to each other with all MCC values between 0.412 and 0.432, and all sensitivity values between 0.771 and 0.786. The closer performance is likely due to the much higher similarity of the targets as characterized by the amino acids descriptor sets. Hence smaller differences are present between targets and modeling performance of descriptor sets will be closer together. Furthermore, the descriptor sets on the protein side now describe a smaller part of the entire data set as we also have the presence of chemical descriptors, which are held constant in the different models.

The best performance has been obtained in this case by the Z-scales (3) and Z-Scales (3) combined with Z-Scales (Avg) (MCC 0.432 and sensitivity 0.786), followed by Z-Scales (Binned), FASGAI, and ProtFP (Feature) combined with Z-Scales (3), (MCC 0.431 and sensitivity 0.786). On the other hand, T-Scales, and ST-Scales (MCC 0.428 and sensitivity of 0.783) followed by ProtFP (Feature) (MCC 0.412 and sensitivity of 0.771) perform the worst. Another interesting observation is that all descriptor sets performed the best on the Muscarinic Acetylcholine receptor (ACM) 4 receptor and the worst on the histamine H3 receptor (followed by the H4 receptor), irrespective of the protein descriptor set selected (Additional file 1: Figure S4; although absolute differences in performance could be observed). The ACM 4 receptor was also modeled best in the LOSO experiments, where the related histamine H4 receptor was modeled worst as discussed in the following.

### LOSO validation GPCRs

**Figure 4:**
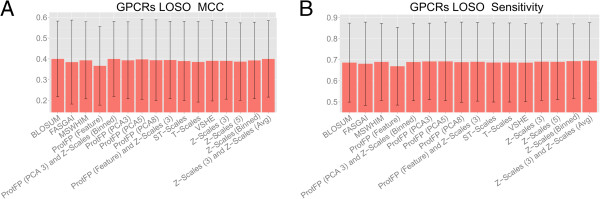
**Mean performance of the benchmarked descriptor sets in the GPCR LOSO validation experiments.** The mean is calculated over all 32 receptors and the error bar represents the standard deviation. Shown are the MCC **(A)** and the sensitivity **(B)**. Note that error bars are large due to different performance between models trained on different GPCRs, not between repeats of the individual models. Here extrapolation takes place on the target side as the test set contains unseen targets. The differences between individual descriptor sets are small. Again for individual receptors larger performance differences occur (see main text and Additional file 1: Figure S11 for details). In this case, Z-Scales (3) and Z-Scales (Avg) is the descriptor set exhibiting best performance while ProtFP (Feature) performs the worst.

In order to benchmark the extrapolation capabilities of the descriptor set a Leave-One-Sequence-Out experiment was performed on the GPCR dataset, the results of which are shown in Figure 4. The overall performance is worse compared to the 70–30 benchmark (MCC values between 0.367 and 0.400 and sensitivity between 0.669 and 0.695). This is to be expected as leaving out a GPCR at the time leaves out a much larger and congeneric part of the data set compared to leaving out a randomized fraction. However there are still some differences that can be observed between the descriptor sets, with the best performance now being delivered by Z-Scales (3) and Z-Scales (Avg) (MCC 0.400 and sensitivity 0.695), followed by the ProtFP (PCA3) and Z-Scales (Binned) (MCC 0.400 and sensitivity 0.689), and BLOSUM (MCC 0.400 and sensitivity 0.686). The worst performance in this experiment is observed for ProtFP (Feature) (MCC 0.367 and sensitivity 0.669); yet it should be noted that the differences are overall relatively small.

Interestingly, the receptor that is modeled the best is again the ACM 4 receptor and the worst is now the histamine H4 receptor (followed by the H3 receptor), irrespective of the protein descriptor set selected (Additional file 1: Figure S5). To gain a further understanding of this constant good performance for the ACM 4 receptor and bad performance of the two Histamine receptors, a PCA analysis was performed analogously to the ACE inhibitors, but then applied to the GPCR binding site sequences.

### Analysis of GPCR target space

**Figure 5:**
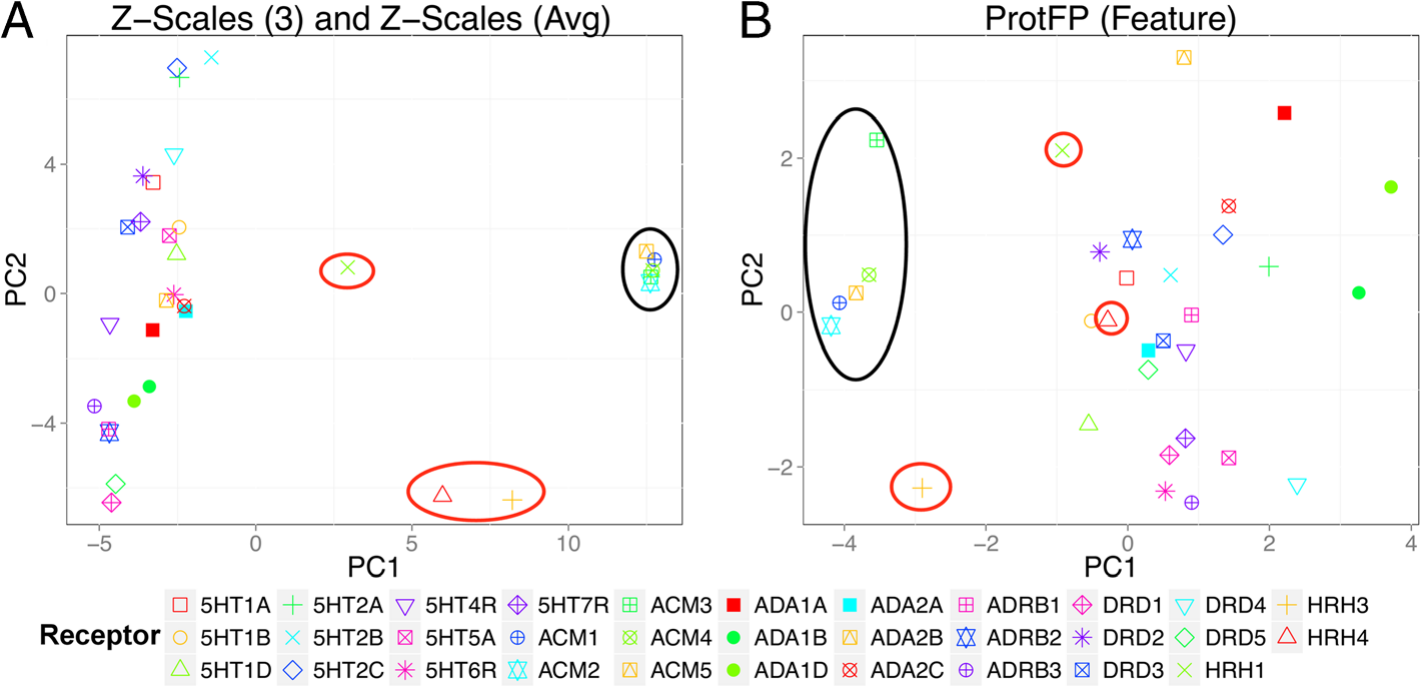
**PCA plot of GPCR data set target space. (A)** Shown are the best performing descriptor (Z-Scales (3) and Z-Scales (Avg)), and **(B)** the worst performing descriptor (ProtFP (Feature)). The **(A)** panel shows are more explicit clustering compared to ProtFP (Feature) in **(B)**. The red circles indicate the histamine receptors and the black circles the muscarinic acetylcholine receptors. The lower performance of the histamine receptor family can be rationalized in both cases, as no clear clustering is apparent for this family. Conversely, both plots demonstrate clustering for the ACM receptors, which might explain the good performance.

From the PCA analysis of target space we can rationalize the poor performance on the histamine receptors (Figure 5, and Additional file 1: Figures S6, S7, S8). In the PCA of all GPCR targets used in this dataset, and employing the different descriptor sets, the histamine receptors are not clustering. The distance between the receptors is rather large, in particular when comparing these distances with the distances between the other receptor families. In literature it has been shown that the chemical space for the ligands of the H1, H3, and H4 receptors actually overlaps (where the similarity between H3 and H4 is higher than with H1) [33,34]. This is only partially reproduced in our target similarity. It is therefore likely that the models are unable to reliably extrapolate for this receptor based on the H1 receptor. Leaving out one receptor removes crucial information that cannot be compensated for by the other two histamine receptors using the current protein description. Therefore the current binding site selection is likely insufficient to accurately model the full target space as was selected here. It is likely that the removal of gapped positions in the alignment at least partially contributes to the observed lack of clustering in the histamine receptor family, in particular as the histamine H1 receptor crystal structure was also used to select the binding site residues (see Methods). While outside the scope of the current paper, an interesting follow up study can be the recursive residue addition / elimination from the binding site selection. This way it can be studied when histamine receptor clustering does appear and to what extend the current selection is insufficient. Additionally this follow up experiment can serve to study possible methods to include gapped amino acid positions. In the current study these were not considered to keep the benchmark fair and level and avoid the introduction of another point of variability (see Methods for further information).

Conversely, other receptor subtypes (5HT, alpha-adrenergic, beta-adrenergic, and dopamine receptors) cluster together, which hence allow leaving one receptor out while still retaining much information about the receptor space of that particular protein family. The well-performing ACM 4 receptor is located in a clear muscarinic acetylcholine receptor subfamily cluster. Leaving this receptor out can therefore be considered straightforward as the target space is well covered. Hence, by analyzing distances of receptors to their nearest neighbors in target space allows rationalization (and also prospective anticipation) of the performance of PCM models based on a set of related receptors.

### Conclusions for GPCRs and ligands

It can be concluded that all different descriptor sets can be used to create predictive PCM models on this set while showing an order of descending performance as follows: Z-Scales (3) and Z-Scales (Avg), ProtFP (Feature) and Z-Scales (3), Z-scales (3), ProtFP (PCA5), Z-Scales (Binned), BLOSUM, and ProtFP (PCA8). The worst 3 are (in again descending order) T-Scales, ST-scales, and ProtFP (Feature). It is striking to see that the combination of Z-Scales (3) with another type of descriptor set (Z-Scales (Avg) or ProtFP (Feature)) actually has a synergistic effect where the combination performs better than the individual sets. However this is not the case for the combination ProtFP (PCA3) and Z-Scales (Binned), which is perhaps caused by the fact that these two sets are very similar.

Furthermore it can be concluded that the binding site definition used for the GPCR descriptors is not optimal for all receptors. While the dopamine, 5HT, muscarinic acetylcholine, alpha adrenergic, and beta adrenergic receptors are modeled very well (and interpolation between receptors works relatively well), the histamine receptors clearly show less ideal performance. It would therefore be advisable to model these receptors with a different binding site definition, a starting point could be the work by Surgand *et al.*[35].

### 70–30 validation on NNRTIs

**Figure 6:**
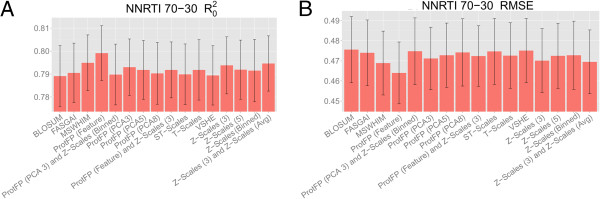
**Mean performance of the benchmarked descriptor sets in the NNRTIs 70–30 validation experiments.** The mean is calculated over all 14 mutants (performed 10 times) and the error bar represents the standard deviation. Shown are the R_0_^2^**(A)** and the RMSE **(B)**. (See Additional file 1: Figure S15 for details.) Slightly more variance is seen compared to the GPCR experiments. In this case BLOSUM performs the worst among all descriptor sets considered, while ProtFP (Feature) performs the best.

While the above GPCR ligand dataset was based on rather diverse ligands, the NNRTI dataset employed in this study covers a more neatly defined area of both chemical (ligand) space as well as biological (target) space and hence we also included the set. Moreover, this set has been very difficult to model with QSAR approaches (leaving out the target information, see methods for further details) and is hence a very good example of cases where PCM can add value [28]. The first step to evaluate descriptor set performance on this set was again a 70–30 validation experiment to assess the ability of the different descriptor sets to capture the ligand–target interaction space, the results of which are shown in Figure 6. Similar to previous experiments on the GPCR set, the performance of the descriptor sets is closely together, with RMSEs in the range 0.464 – 0.476 and R_0_^2^ in the range 0.789 – 0.799. However, in this set the ProtFP (Feature) performs the best (RMSE 0.464 and R_0_^2^ 0.799), followed by MSWHIM (RMSE 0.469 and R_0_^2^ 0.795) and Z-Scales (3) combined with Z-Scales (Avg) (RMSE 0.470 and R_0_^2^ 0.795). The worst performance comes from (descending) ProtFP (PCA3) combined with Z-Scales (Binned) (RMSE 0.475 and R_0_^2^ 0.790), VHSE (RMSE 0.475 and R_0_^2^ 0.789), and BLOSUM (RMSE 0.476 and R_0_^2^ 0.789).

When focusing on the individual mutants (Additional file 1: Figure S9), the best performing mutants are either sequence 12 (K101E and K103N) and 9 (K103N), both covered well in the remaining training set. Most descriptor sets are able to model the fraction of the compounds left out with an RMSE of <0.3 log units on these mutants. The mutants that are modeled the worst are surprisingly not the heavy mutant sequence 7 (which contains a number of 13 total mutations), but rather sequence 2, and 6. Sequence 2 is carrying only two mutations (V179F and Y181C), where V179F is known to have a high impact on the class of compounds modeled here and the mutation itself (from valine to phenylalanine) is also a large change with respect to the physicochemical properties of the residues involved [36]. Furthermore, this mutation was identified as having the most effect on binding in previous work, which is consistent with the current observations [28]. Sequence 6 is the only sequence containing the E138G mutation and can be considered a singleton (also modeled badly in previous work) [28]. Still it should be noted that even in the case of the poorly modeled mutant, there are individual differences between descriptor sets (with the RMSE ranging between 0.575 and 0.644, and the R_0_^2^ ranging from 0.314 to 0.404). Subsequently it was investigated whether results were transferable to the LOSO experiment, when extrapolation abilities to entirely novel sequences were required.

### LOSO validation on NNRTIs

**Figure 7:**
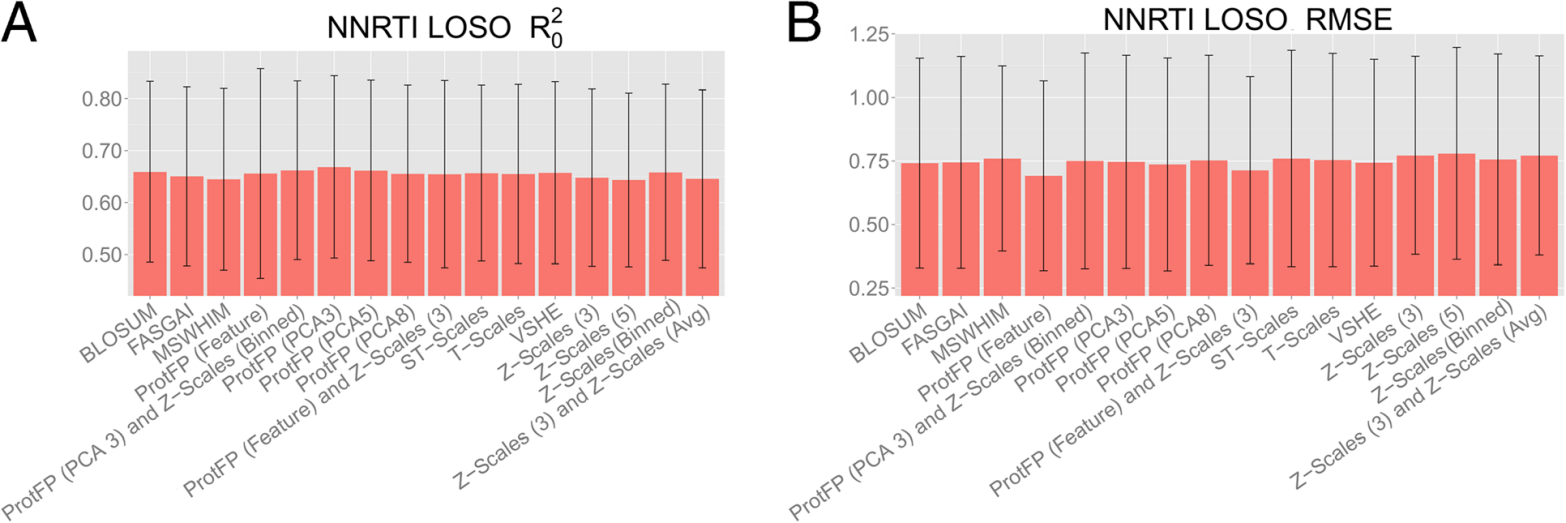
**Mean performance of the benchmarked descriptor sets in the NNRTIs LOSO validation experiments.** The mean is calculated over all 14 mutants and the error bar represents the standard deviation. Shown are the R_0_^2^**(A)** and the RMSE **(B)**. Note that error bars are large due to different performance between models trained on different mutants, not between repeats of the individual models. Extrapolation takes place on the target side as the test set contains unseen targets. The differences between individual descriptor sets are still small but the spread of the standard deviation increases. Again for individual receptors larger performance differences occur (see main text and Additional file 1: Figure S10 for details). In this part of the study ProtFP (Feature) shows very good performance, which indicates that a simplified representation on the protein side is favorable for this data set.

The LOSO validation was performed in a similar manner to the GPCR LOSO validation, leaving out one sequence at a time from model training, and predicting the activity of compounds on the sequence that was left out. The results are shown in Figure 7. The best performance can be observed for ProtFP (PCA5) (RMSE 0.736 and R_0_^2^ 0.662) followed by ProtFP (PCA3) (RMSE 0.747 and R_0_^2^ 0.668), BLOSUM (RMSE 0.741 and R_0_^2^ 0.659), and ProtFP (Feature) (RMSE 0.736 and R_0_^2^ 0.662). The worst performance is obtained by (in descending order) Z-Scales (3) and Z-Scales (Avg) (RMSE 0.771 and R_0_^2^ 0.646), MS-WHIM (RMSE 0.760 and R_0_^2^ 0.645), and Z-Scales (5) (RMSE 0.779 and R_0_^2^ 0.644). Noteworthy is that, while the mean RMSE rises to 0.779 log units (calculated over all sets), the mean R_0_^2^ (calculated over all sets) remains relatively high (at values larger than 0.64). This indicates that the descriptor sets are introducing a consistent offset in the predictions, while still in most cases being able to accurately rank the compounds relative to each other. Indeed it could be considered logical that compound ranking depends more on compound descriptors (where in this case ECFP_6 fingerprints were employed), which were kept constant In PCM modeling predictions are always dependent on both descriptors types.

The mutants that performed the best in the models were again sequence 12 (carrying solely the K101E and K103N mutation) as well as sequence 3, (carrying solely the Y181C mutation). Both are very well covered in the training set. The sequence modeled the worst was again sequence 6 (carrying the E138G mutation). This sequence was also most difficult to model in LOSO in previous work [22], and as mentioned above the cause is likely that this sequence forms a singleton as it is the only sequence carrying mutation E138G. It is striking that ProtFP (Feature) performs significantly better on this data set than the other two sets. On the NNRTI set, ProtFP (Feature) ranks 1st in the 70–30 validation and 4th in the LOSO validation, in the ACE inhibitor set it ranks 16th and also in the GPCR set the descriptor ranks 16th. A PCA analysis was again performed to connect these observations of descriptor set performance to the similarity of the sequences and the way the descriptor sets characterize the space.

### Analysis of NNRTI target space

**Figure 8:**
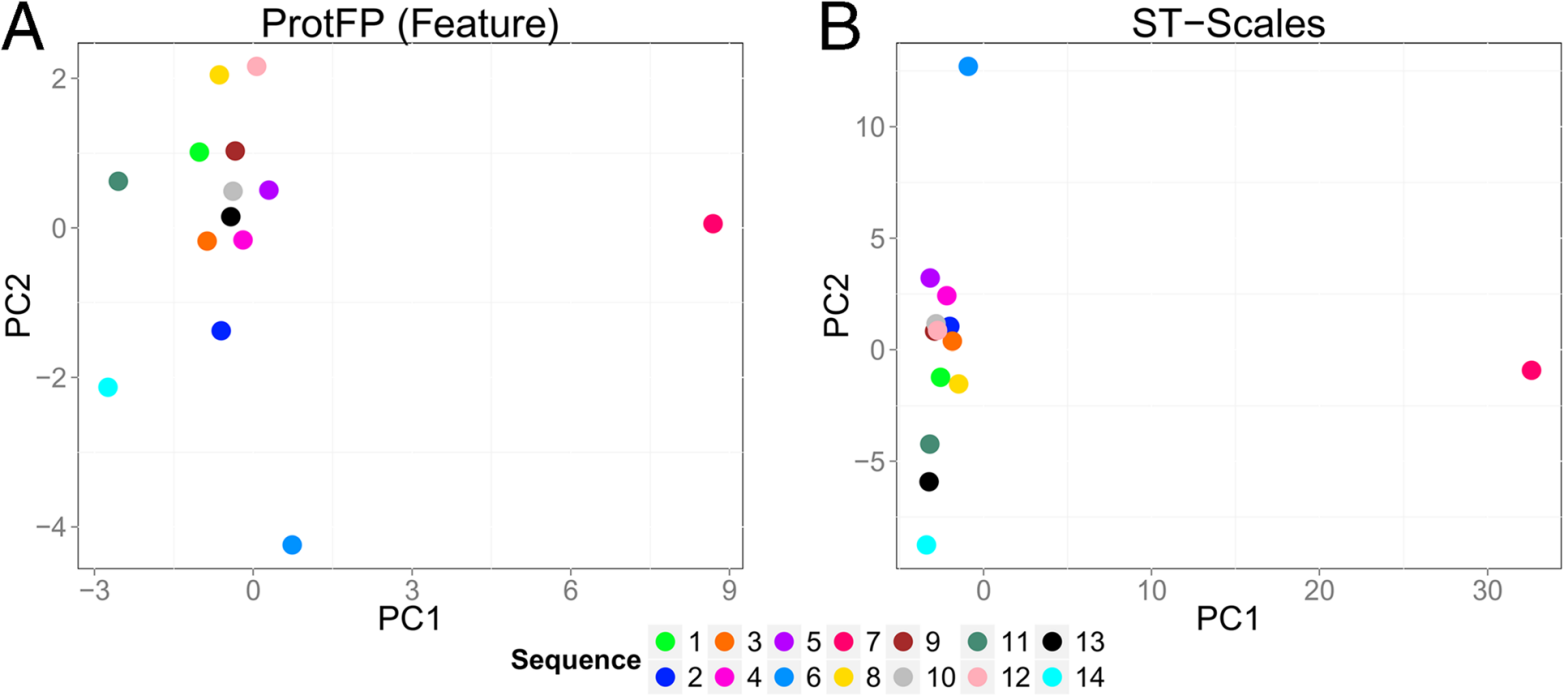
**PCA plots of the best and worst performing descriptor sets on the NNRTI benchmark. (A)** The simplified representation of ProtFP (Feature) proves to be an advantage on this congeneric data set as the distance in PCA space better correlates to the distance in bioactivity space. **(B)** The ST-Scales on the other hand perform the least well, and it can be hypothesized that the tight clustering in one part of the plot does not correlate to bioactivity space.

The PCA analysis can explain the better performance of ProtFP (Feature) (Figure 8, Additional file 1: Figures S11, S12, S13) given the following findings. Due to the fact that the mutants only differ by point mutations and one of the sequences caries 13 mutations (sequence 7), this sequence is set far apart from the other sequences by most descriptor sets. This effect is much less pronounced in ProtFP (Feature) as it does not differentiate between the type of mutations (all AAs are encoded as features so every amino acid difference is equal). At the same time, a number of sequences only differ by a single amino acid that can be very similar (e.g. leucine to isoleucine). These differences are maximal in the case of ProtFP (Feature) whereas they are relatively small in the case of Z-Scales (5). The combined effect is that all sequences cluster much more evenly distributed throughout the PCA space using ProtFP (Feature) compared to the other descriptor sets, leading to a better performance on this particular set. While this effect proves beneficial in this particular case where chemical and target space are closely defined, it should be noted that this is no guarantee to be a general effect, as can be seen on the other bioactivity benchmarks where ProtFP (Feature) performs below average. Another cause for the observed effect could be the following. By leaving out the residues that did not mutate in any of the sequences, the focus was on the sequence dissimilarities. Hence the descriptor representation of the target similarity does not accurately represent the overall still quite large similarities in bioactivity space. As the ProtFP (Feature) descriptor set leads to relatively small distances by merely encoding the presence or absence of a feature, it partially compensates for the distorted representation of target similarities.

### Conclusions for NNRTIs and mutants

The NNRTI set represented a different data set compared to the GPCR ligand dataset evaluated above, as it consists of a number of highly similar sequences and compounds and, hence, resembles a typical data set one might encounter in lead optimization. It is concluded that in these cases the feature-based descriptor set performs very well; however its good performance can also be caused by our binding site definition. Therefore this type of descriptor set should be included as a possible candidate when working on a data set consisting of several highly related targets. However, as shown in the other benchmarks this is not an observation that can be generalized to all datasets.

The final ranking for this dataset is as follows: The best performing feature set was ProtFP (Feature), followed by, ProtFP (PCA3), ProtFP (PCA5) (both of which exhibit similar performance), MS-WHIM, ProtFP (Feature) and Z-Scales (3). The worst performing descriptor sets were found to be (in descending order) ProtFP (PCA3) and Z-Scales (Binned), Z-Scales (5) and ST-Scales.

### 70–30 validation on PIs

**Figure 9:**
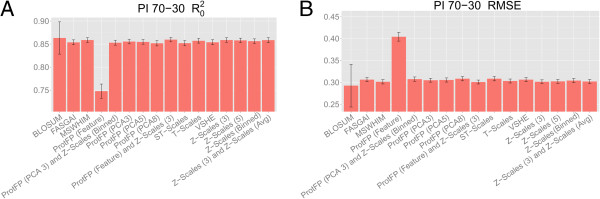
**Mean performance of the benchmarked descriptor sets in the PIs 70–30 validation experiments.** The mean is calculated over all repeats (performed 10 times) and the error bar represents the standard deviation. Shown are the R_0_^2^**(A)** and the RMSE **(B)**. Slightly more variance between descriptor sets is seen compared to the GPCR experiments and NNRTI experiments. In this case ProtFP (Feature) performs the worst among all descriptor sets considered, while BLOSUM performs the best.

The last benchmark data set was a set consisting of clinically approved inhibitors of HIV Protease and a very large set of mutants (1060 full sequences). Again the performance of the protein descriptor sets is compared in both a 70–30% validation and LOSO approach. The results are shown in Figure 9 and again differences are negligible. The best performing descriptor is BLOSUM (RMSE 0.293 and R_0_^2^ 0.863), followed by ProtFP (Feature) combined with Z-Scales (3) (RMSE 0.301 and R_0_^2^ 0.860), and MS-WHIM (RMSE 0.301 and R_0_^2^ 0.859). The worst performing are in descending order: ProtFP (PCA8) (RMSE 0.308 and R_0_^2^ 0.852), ST-Scales (RMSE 0.308 and R_0_^2^ 0.852), and ProtFP (Feature) (RMSE 0.404 and R_0_^2^ 0.748). It is noteworthy to see here that the combination of the worst performing ProtFP (Feature) combined with Z-Scales (3) again has a synergistic effect. The combined descriptor sets perform better than only the Z-Scales (3). It could be caused by ProtFP (Feature)’s abilities to pick up point mutations (as shown in the NNRTI section), however containing too little information to accurately describe the bioactivity space (as shown by the bad performance of ProtFP (Feature) alone).

### LOSO validation on PIs

The LOSO experiment was performed slightly different on this dataset. Given the very large size and the computational infeasibility to repeat this for 16 descriptor sets, not a single target was left out but 10% at a time. From earlier work it was known that this can indeed be done and that the results are still comparable; the key issue is to leave out a full section of the target space with all bioactivity annotations. [7] Still, this corresponds roughly to leaving out a single target out of 14 in the NNRTI set (+/− 10% of target variation).

**Figure 10:**
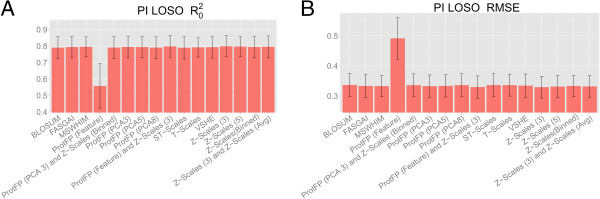
**Mean performance of the benchmarked descriptor sets in the PIs LOSO validation experiments.** The mean is calculated over all mutants (leaving out 10% at a time) and the error bar represents the standard deviation. Shown are the R_0_^2^**(A)** and the RMSE **(B)**. Again for individual targets larger performance differences occur (see main text for details). In this part of the study ProtFP (Feature) performs poorly, while it performs very well when paired with Z-Scales (3). The best performance is by Z-Scales (3).

The results of this experiment are shown in Figure 10. Best performing was now the Z-Scales (3) set (RMSE 0.329 and R_0_^2^ 0.800), followed by ProtFP (Feature) combined with Z-Scales (3) (RMSE 0.330 and R_0_^2^ 0.799), and the Z-Scales (5) set (RMSE 0.331 and R_0_^2^ 0.798). Worst performing were ST-Scales (RMSE 0.336 and R_0_^2^ 0.790), BLOSUM (RMSE 0.337 and R_0_^2^ 0.790), and ProtFP (Feature) (RMSE 0.492 and R_0_^2^ 0.558). However, all descriptors perform very well on this high quality set with very little difference. In order to rationalize performance differences a PCA was performed.

### Analysis of PI target space

**Figure 11:**
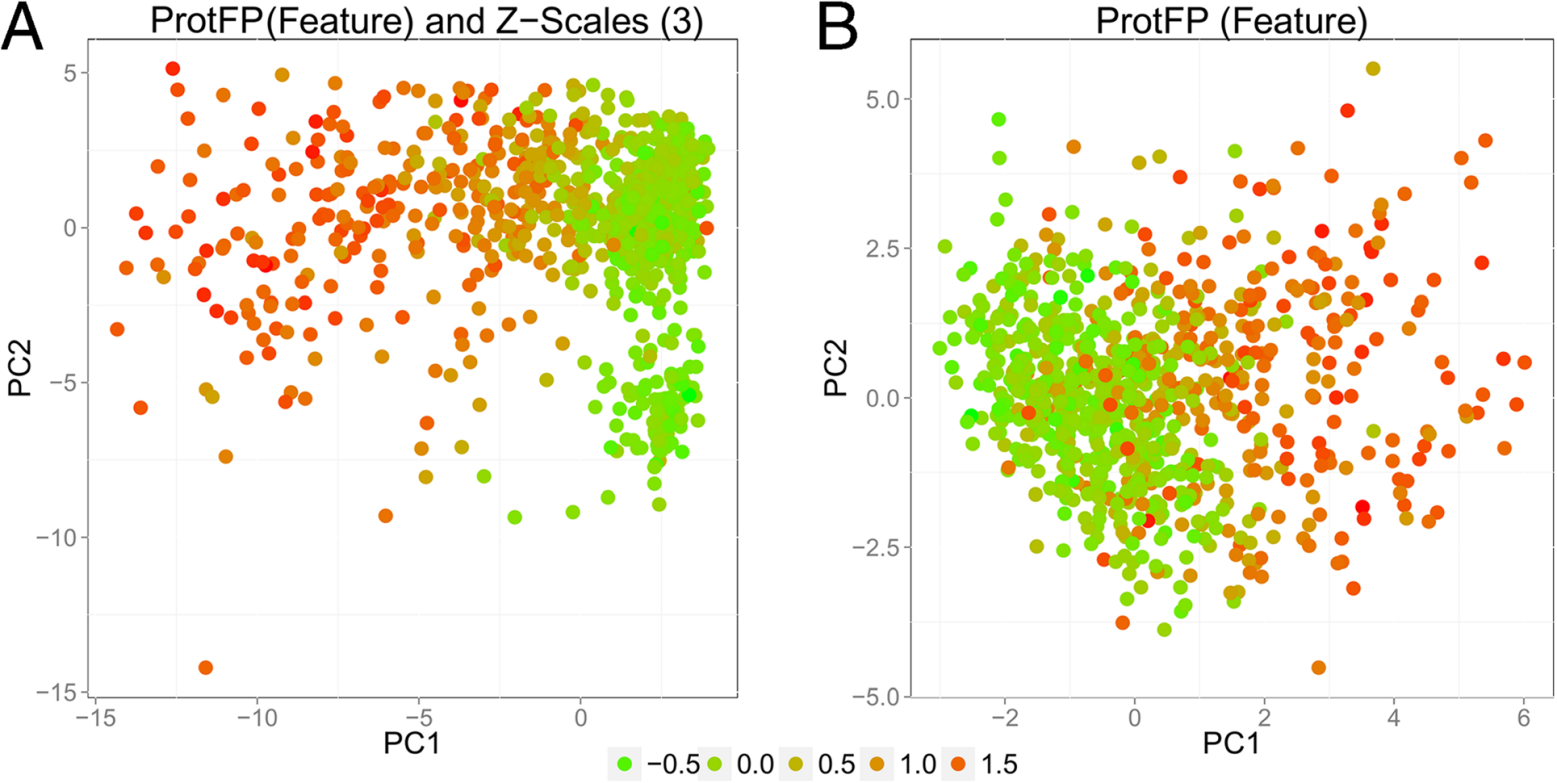
**PCA plots of target similarity of the protease mutants.** Shown are **(A)** the best and **(B)** worst performing descriptor sets. The feature based descriptor only codes for presence or absence of features. This leads to points scattered over a smaller area in PCA space and could explain the decreased performance **(B)**. However, the information is shown to have a synergistic effect when combined with a physicochemical property based descriptor **(A)**.

In order to get an insight into the descriptor performance we performed a PCA analysis on target space again (Figure 11, Additional file 1: Figures S14, S15, S16). The points were colored by average resistance to visualize the bioactivity space as the number of sequences was too large for individual coloring. The PCA plots confirm the results seen in both 70–30 validation and LOSO validation. Most descriptors are able to separate the mutants in a way that correlates to their activity. The exception is ProtFP (Feature) where the spread of the sequences in PCA spaces seems clustered over a smaller space and there is overlap between mutants with low resistance (green) and higher resistance (red). This could be caused by the fact that this descriptor merely considers the amount of point mutations (were the distance between two diverse amino acids is identical to the distance between two similar amino acids). However, when this is combined with the similarity-based amino acid distance provided by the Z-Scales (3), both descriptor sets appear to be synergistic, leading to a slight performance improvement.

### Final descriptor set ranking

**Figure 12:**
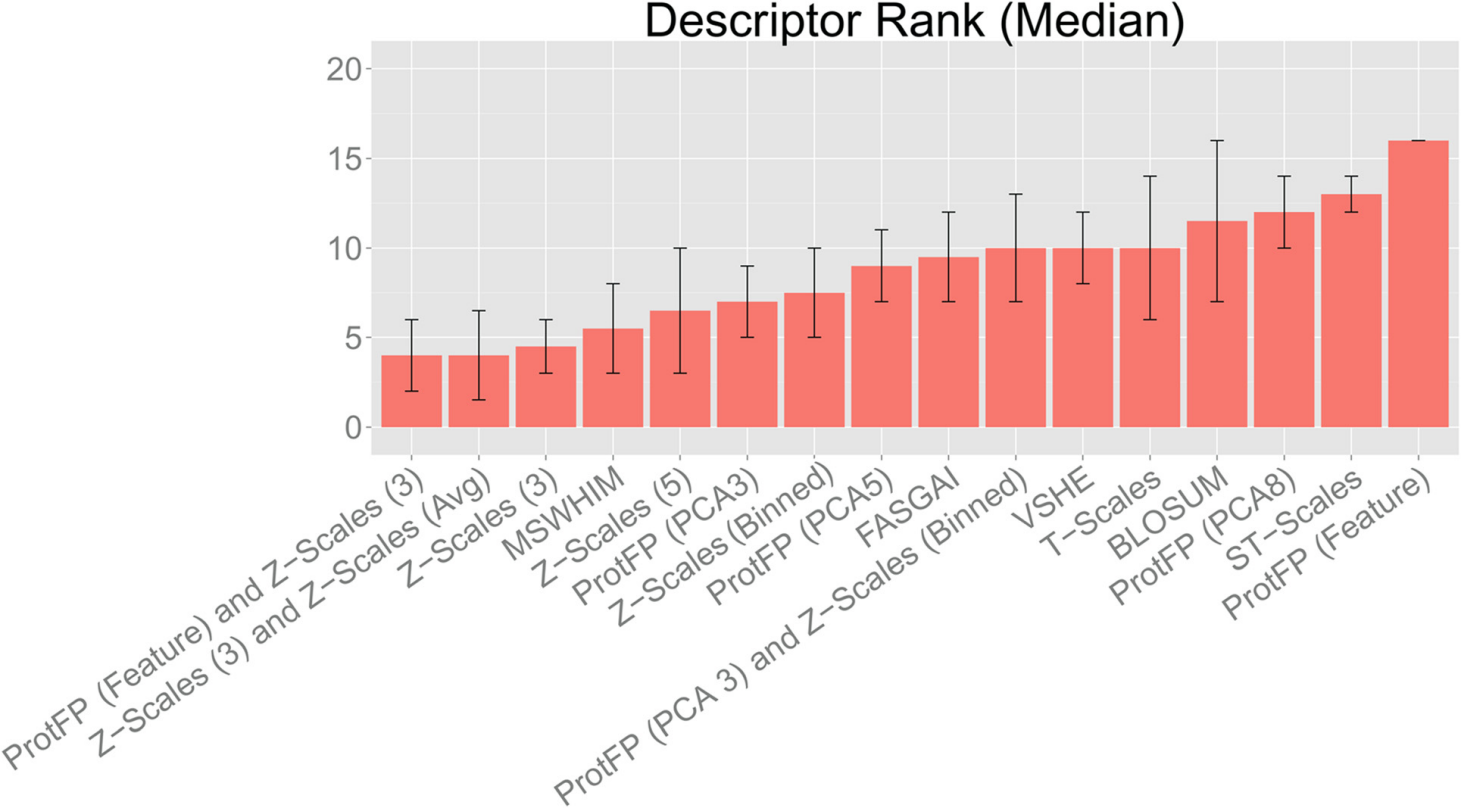
**Median rank of the descriptor sets in the bioactivity benchmarks.** The median is calculated over all 14 ranks (1 rank per dataset, per experiment, per validation type), also shown the median average deviation (MAD). The best three descriptor sets have a median rank < 5 among which the combinations of Z-scales (3) with other descriptors perform the best. The worst performance is by BLOSUM, ProtFP (PCA8), ST-scales and ProtFP (Feature) with a mean rank > 11. BLOSUM has a large standard deviation due to its inconsistent performance.

The final ranking of the individual descriptor sets is given in Table 3 and Figure 12. The table included the individual ranks of all descriptor set in each experiment (on a scale of 1 to 16) and a final overall ranking (which is calculated as the median of the individual rankings). Also included is the median average deviation of the median (MAD) of the median rank in this representation (Figure 12). In the discussion of the following it should be kept in mind that differences in performance were on most datasets rather small, so that differences in ranks usually translate to small quantitative differences in performance.

**Table 3:** Overall descriptor set ranking

**Type**	**Final rank (MAD)**	**Rank ACE**	**Rank GPCR**	**Rank NNRTI**	**Rank PI**
ProtFP (Feature) and Z-Scales (3)	4 (±2.0)	8.5	4	7.5	2
Z-Scales (3) and Z-Scales (Avg)	4 (±2.5)	11	1	8.5	4.5
Z-Scales (3)	4.5 (±1.5)	6	5	8.5	2.5
MS-WHIM	5.5 (±2.5)	4	8.5	7	4
Z-Scales (5)	6.5 (±3.5)	3	12.5	11.5	4.5
ProtFP (PCA3)	7 (±2.0)	10	9	5	7.5
Z-Scales (Binned)	7.5 (±2.5)	1.5	6.5	10	7.5
ProtFP (PCA5)	9 (±2.0)	15	5.5	5	9.5
FASGAI	9.5 (±2.5)	6.5	9.5	11	9.5
ProtFP (PCA3) and Z-Scales (Binned)	10 (±3.0)	1.5	8	11	12.5
T_Scales	10 (±2.0)	8.5	13	9	9
VHSE	10 (4.0)	5.5	9	10.5	10.5
BLOSUM	11.5 (±4.5)	13.5	8	10	7.5
ProtFP (PCA8)	12 (±2.0)	13.5	8	10.5	13.5
ST_Scales	13 (±1.0)	12	13.5	13	14.5
ProtFP (Feature)	16 (±0.0)	16	16	1	16

The descriptor sets are sorted based on their median (final) rank calculated over all 14 benchmarks and the MAD associated with this value. Also shown is the rank each descriptor set receives in each individual data set (calculated over the four benchmarks per data set). The best performance is achieved by combining the Z-scales (3) with complementary descriptor types (ProtFP (Feature) or Z-scales (Avg)), closely followed by Z-Scales (3), MS-WHIM, and Z-Scales (5).

The best performing descriptor sets overall are, ProtFP (Feature) combined with Z-Scales (3) (median rank 4.0 (±2.0)), Z-Scales (3) combined with Z-Scales (Avg) (median rank 4.0 (±2.5)), Z-Scales (3) (median rank 4.5 (±1.5)), MSWHIM (median rank 5.5 (±2.5)), Z-Scales (5) (median rank 6.5 (±3.5)), and ProtFP (PCA3) (median rank 7 (±2.0)). Following from the results it is apparent that combining Z-Scales (a physicochemical descriptor) with complementary information leads to synergy, see below for a further discussion on this observation.

The worst performing descriptor sets are ProtFP (PCA8) (median rank 12 (±2.0)), ST-scales (median rank 13 (±1.0)), and ProtFP (Feature) (median rank 16 (±0.0)). While their performance was sometimes close to the other descriptor sets, they were found among the lower performing ranks in 80% of the experiments. Therefore it might be wise to avoid these descriptor sets on bioactivity modeling in setups such as the PCM modeling employed here; but this again will surely depend on the particular dataset at hand as well (for example a data set similar to the NNRTI set in the case of ProtFP (Feature)).

### Usage of more than 3 principal components per amino acid

In the companion publication of this work it was observed that using or more principal components per amino acid leads to a large shift in descriptor set behavior (characterized as the way in which a descriptor set perceives amino acid similarity) [21]. Yet including the 4th or even 8th principal component only leads to a marginal increase in percentage explained of the variance from the original matrix. Here it is observed that in the cases where multiple components are included (e.g. Z-Scales (5) as opposed to Z-Scales (3)), the performance decreases and in most cases using fewer components leads to better performance. Indeed the top 6 performing descriptor sets only contain 1 set with more than 3 components whereas the lowest performing 6 contain no set with less than 5 components. Hence we would conclude that using more that 3 PCs per amino acids leads to overtraining in the case of PCM models, corroborated by descriptor sets performing well in the 70–30 validation and then poorly in LOSO. It would be advised to use less information per amino acids.

### Statistical significance of descriptor differences

In order to support these observations we have performed a statistical test on all pairs of descriptor sets to investigate whether differences in performance were significant. As a T-test assumes a normal distribution of the data we could not apply it here, instead we applied a Wilcoxon signed rank test [37]. Table 4 lists the complete matrix of p values when the descriptors are compared. The most important observation is that ProtFP (Feature) is significantly worse than all others (p < 0.05) with the exception of Z-Scales (5). Moreover while combining Z-Scales (3) with other descriptor types proves to be better, this difference is not significant (p = 0.78 for the combination with ProtFP (Feature) and p = 0.64 for the combination with Z-Scales (Avg)).

**Table 4:** Overview of significance of differences between descriptor ranks

**Descriptor**	**ProtFP (Feature) and Z-Scales (3)**	**Z-Scales (3) Z-Scales (Avg)**	**Z-Scales (3)**	**MS-WHIM**	**Z-Scales (5)**	**ProtFP PCA (3)**	**Z-Scales (Binned)**	**ProtFP PCA (5)**	**FASGAI**	**ProtFP PCA (3) and Z_scales (Binned)**	**VHSE**	**T-Scales**	**BLOSUM**	**ProtFP PCA (8)**	**ST-Scales**
Z-Scales (3) and Z-Scales AVG	1.00														
Z-Scales (3)	0.78	0.64													
MS-WHIM	0.27	0.34	0.56												
Z-Scales (5)	0.05	0.10	0.17	0.47											
ProtFP PCA (3)	0.09	0.18	0.15	0.50	0.78										
Z-Scales (Binned)	0.26	0.38	0.29	0.89	0.63	0.78									
ProtFP PCA (5)	0.02	0.15	0.17	0.30	0.80	0.78	0.37								
FASGAI	0.01	0.03	0.03	0.15	0.56	0.13	0.10	0.46							
ProtFP PCA (3) and Z_scales (Binned)	0.05	0.17	0.17	0.42	0.82	0.23	0.15	0.78	0.98						
VHSE	0.01	0.02	0.02	0.08	0.49	0.15	0.09	0.31	0.95	0.82					
T-Scales	0.00	0.01	0.01	0.03	0.13	0.02	0.01	0.15	0.56	0.80	0.58				
BLOSUM	0.10	0.14	0.19	0.35	0.71	0.34	0.27	0.56	0.87	0.61	1.00	0.98			
ProtFP PCA (8)	0.00	0.01	0.00	0.01	0.09	0.00	0.00	0.04	0.16	0.25	0.22	0.26	0.75		
ST-Scales	0.00	0.00	0.00	0.00	0.02	0.00	0.00	0.00	0.00	0.01	0.01	0.00	0.24	0.08	
ProtFP (Feature)	0.01	0.01	0.01	0.02	0.05	0.02	0.02	0.03	0.03	0.02	0.03	0.03	0.04	0.04	0.05

Calculated over all 14 ranks using a Wilcoxon signed rank test., given is the p-value for a paired comparison.

### Complementary protein descriptors

Most descriptor sets have been found to perform very similar (no statistical differences), yet this not very surprising as the majority of the descriptors used here have previously been published and hence been validated. Moreover, in literature other studies have appeared applying PCM (although named differently) on GPCR data sets similar to our set here. This includes both the use of feature based descriptor sets [38] and physicochemical descriptor sets [9,18,39]. Performance is similar to the performance found here on the GPCR data set. From literature it can be concluded that using physicochemical properties leads to better performance than feature based descriptors (at least on GPCRs), which is in line with the results of this work. However, it is striking to see that in the current work, in both cases where Z-Scales (3) was combined with another descriptor set (namely the average sequence values (AVG) and ProtFP (Feature)), this combination performed better than only Z-Scales (3). Even the last scoring ProtFP (Feature) appears to add complementary information. While we did not find these results to be significant, the results were consistent. Therefore it could also be the case that just coding for amino acid similarity, as is common in literature, while predictive ignores relevant information that is present in the protein structure. Perhaps other sources of information should be included and one start could be the incorporation of protein flexibility or secondary structure that could be retrieved from nearest neighbor crystal structures or homology models [40,41].

Another cause for this observed close performance could be, as we mentioned in the GPCR section, that the binding site selection used here is not optimal, yet in literature with different binding site selections similar performance is reached [9,18,39]. In this respect it would be interesting to investigate the use of non-alignment dependent descriptors such as PROFEAT [42], CTD [43], or descriptors from the PROPY package [44].

A final option could be the usage of chemogenomics based descriptors. These can consist of phylogenetic trees which are generated based on the tanimoto similarity of ligands known for each protein (via the Similarity Ensemble Approach) [45,46].

### Training times

One final property of the descriptor sets has not been highlighted yet. On a workstation with a core i7 860 CPU and 16 GB memory, considerable differences in training times were found for the individual descriptor sets. On the datasets used in this work, as a rule of thumb ProtFP (Feature) showed the fastest model training while BLOSUM required most time (on average 191% of the training time required for ProtFP (Feature). The reason for this large difference is likely that the feature based descriptor set uses a single variable per amino acid, where the numerical descriptor sets use 3 (ProtFP (PCA3), Z-scales (3) and MS-WHIM) to 10 values (BLOSUM).

## Experimental

All models, with the exception of the timed runs, were trained and validated on the EBI cluster, for further details please see Methods section below. Included in the supporting information (Additional file 2) is a pipeline pilot protocol that allows the transfer of single letter amino acids sequences into the here benchmarked descriptor sets.

## Conclusions

Overall performance differences between amino acid descriptor sets used in this study were rather small, with differences in the order of RMSE differences between 0.01 – 0.1 log units. Hence, as a first approximation – and with some differences between datasets - all descriptor sets considered in this study can be used to train predictive PCM models. Yet, a number of descriptor sets were observed to consistently score good, namely Z-Scales (3) combined with Z-Scales (Avg), ProtFP (Feature) combined with Z-Scales (3), Z-Scales (3), MS-WHIM, Z-Scales (5), and ProtFP (PCA3).

Performance on different targets exhibits significantly larger differences in performance than differences between descriptor sets; for example the RMSE difference between the HIV mutant modeled best and worst was 1.2 log units. Hence, attention still needs to be paid to the question of whether a particular descriptor set is suitable for the protein target to be included in a particular model.

Combining descriptor sets, such as the feature-based ProtFP (Fature) with the physicochemical property-based descriptor set Z-Scales (3), small but consistently improved model performance, which is likely due to the different way these descriptor sets characterize amino acids. This observed effect is conceptually similar to circular fingerprints (also feature based) being complementary to physicochemical small molecule descriptors as we observed in the ligand descriptors.

Hence we would recommend the use of the Z-Scales (3) (possibly combined with a feature based fingerprint) for applications in proteochemometric models. On the other hand there are 3 descriptor sets that consistently score less well on the datasets used here, namely ProtFP (PCA8), ProtFP (Feature), and ST-Scales. Based on the information available, these would be less ideal for use in PCM models.

## Methods

For a more detailed description of each descriptor set, both the way they are derived and the extent to which they behave similarly and differently, the reader is referred to the previous work. [21]. This methods section will be limited to the methods relevant for obtaining the result described later in the current study.

### Benchmark datasets for the descriptor sets

Analyzing similar and different behavior of AA descriptor sets is relevant to judge *how similarly* two descriptor sets behave as shown previously [21]. However, this analysis does not yet give any information *how relevant* the information captured by a particular descriptor would be for the generation of bioactivity models. Hence, in order to assess the performance of each descriptor set, four different data sets were used to perform a number of benchmark experiments.

### ACE inhibitor data set

The first set consisted of 58 dipeptides with a measured ACE inhibiting effect (pIC_50_) and was obtained from literature [29]. The set serves as a benchmark as several of the descriptor sets analyzed here were applied to this set in their original publication. Hence, it is demonstrated that the method used (Random Forest) performs *on par* or better than the PLS which is conventionally used in QSAM publications (see Additional file 1: Table S1 for the comparison). See Table 2 for further details about the data set.

### GPCR data set

The second bioactivity data set employed for benchmarking different amino acid descriptor sets comprised a subset of 32 human monoamine receptors (class A GPCRs listed in Additional file 1: Table S2; see also Additional file 1: Figure S17 regarding the subset of receptors used) obtained from ChEMBL version 16 [21]. Receptors were selected only if more than 120 unique ligands (and optimally 200) with annotated activity were present in ChEMBL. A binding site residue selection was obtained using the program JOY [47]. All known GPCR structures in the pdb up to December 2012 were superposed and residues in contact with the ligand were selected. Subsequently residues were translated to the aminergic GPCRs counterpart using the alignment from GPCRdb [48]. Residue positions that were gapped in any of the aminergic GPCRs or positions for which no GPCRdb alignment number was available were discarded. These positions were discarded to keep the benchmark fair and level as different methods to incorporate gapped positions might benefit one descriptor set over the others (for instance usage of ‘0’ descriptors might benefit those descriptor sets with a smaller range of continuous values). The alignment is provided as Additional file 3 and the data set can be downloaded from http://www.gjpvanwesten.nl/proteindescriptors. Residues selected were subsequently subject to conversion into numerical values using all protein descriptor sets listed above.

For each of the 32 receptors included in this study all small molecules with an affinity on this receptor available in ChEMBL were selected and further narrowed down to only include Ki annotations with a protein confidence score of 9. Compounds were then classified as ‘active’ (pKi > 7) or ‘inactive’ (pKi = < 7). Finally compounds were clustered (using the ECFP_6 fingerprint, also used to train the models) to obtain a total of 100 chemically diverse ‘actives’ and 75 chemically diverse ‘inactives’ per receptor, in addition a random 25 compound from ChEMBL were included as presumed inactives (based on the work of Heikamp and Bajorath) [49]. Compounds were standardized, salts were removed, and ionized at pH 7.4 in Pipeline Pilot 8.5 [50]. In total 3,230 distinct compounds were selected to generate a bioactivity model, the final dataset comprising 6,046 ligand-protein data points. This corresponds to 6% of the total of 103,360 possible compound–receptor combinations in the full matrix of 3,230 compounds and 32 targets; see Table 2 for further details.

### NNRTI data set

The second bioactivity data set subjected to PCM modeling comprised 14 mutants of HIV Reverse Transcriptase and 451 Non-Nucleotide Reverse Transcriptase Inhibitors (NNRTIs), and hence a total of 6,314 possible compound–receptor combinations out of which for 4,024 a pEC_50_ value was available (66% of the total) [28]. The compounds in this case were structural analogues, and hence (as opposed to the GPCR case) the average similarity between the compounds was higher, as was the similarity between the protein targets (which were mutants carrying between a single and 13 point mutations). Like in previous work, the binding site was defined as those AAs that differed between the different mutants (a total of 24 residues) [28]. The HXB2 / IIIB reference strain was defined as the wild type [51]; see Table 2 for further details.

### PI data set

The third and final PCM set comprised of 1060 HIV protease mutants and 9 clinical protease inhibitors (PIs) [7]. HIV proteases and proteases in general have previously been shown to be amenable to PCM modeling [52-54]. The set consisted of a total of 6,995 bioactivity points, in the form of a pIC_50_ fold change (difference between mutated protein pIC_50_ and wild type pIC_50_), and was hence 73% complete. The compounds were not as similar as in the NNRTI set but at the same time not as diverse as the GPCR set. The full sequence of the protein was used (99 amino acids as it is a dimer) as was done in earlier work [7], hence the target space was considerably more diverse but the average similarity was high. The HXB2 / IIIB reference strain was defined as the wild type [51]; see Table 2 for further details.

### Amino acid descriptor set benchmarking

Two different approaches were pursued to benchmark AA descriptor sets with respect to their ability to generate bioactivity models (and hence, to capture protein information relevant to bioactivity and ligand binding), namely 70–30 validation and Leave-One-Sequence-Out (LOSO) which are described in the following.

### 70–30 validation

The first benchmark employed in this study was a ‘70-30’ validation experiment. Each descriptor set was used in turn in combination with each of the datasets, and a model was trained on a random 70% of the data available and used to predict the bioactivities of the remaining 30% of the data. This procedure was repeated ten times with different random splits and from the resulting validation parameters the mean and standard deviation were calculated. For the ACE inhibitors this represented a particularly challenging benchmark as this set only includes peptides and no small molecules. Hence only the amino acid descriptor sets could be used to characterize similarity for unknown data points whereas in the PCM sets the distance is characterized by the combination of amino acid descriptor similarity and ligand descriptor similarity. For the bioactivity datasets employed for PCM modeling (which takes both ligand-side and protein-side descriptors into account) this benchmark provides an answer to two different questions. Firstly, the model was asked to make bioactivity predictions for those compounds that are not present in the training set and hence to extrapolate in the *chemical domain*. This part of the validation was particularly emphasized in case of the GPCR and PI data sets set due to the relatively lower average compound similarity. Hence the model is asked to extrapolate the activity of known compounds and targets to *unknown compounds*.

Secondly, a compound can be present in the training set as annotated on one target, and also be present in the test set as annotated on target 2. This part of the validation was hence emphasized in case of the NNRTI data set due to the high average compound similarity. In this case the model is asked to extrapolate the activity of known compounds and targets to *unknown combinations of the two*, while, individually, each chemical structure and sequence have been seen by the model before, but just not in this particular combination.

### Leave-one-sequence-out validation

This validation experiment was performed for each *target* in order to assess extrapolation abilities of the PCM models in the biological / target domain. Hence this validation was only applied to the datasets containing targets (GPCR set, NNRTI set, and PI set). In this part of the work, in turn a single target is left out of the training set and subsequently a model is trained on all remaining bioactivity data points. Afterwards activity values of all compounds on the target left out of the initial training procedure are predicted and compared to the experimental values. These steps are repeated for all targets in the data set in turn. The exception was the PI set, here not a single target but 10% of the targets was left out. The reason for this was that performing a 1060-fold LOSO experiment for each descriptor was computationally very demanding. While we have done so successfully in the past, we have learned that for this particular set, comparable results (stressing the descriptors better) could be achieved leaving out 10% rather than one sequence [7]. The important distinction between this set up and the 70–30 models is that *sequences and all their annotated bioactivities* are left out of the training set.

This type of validation is a specialty of PCM modeling since it takes advantage of its ability to extrapolate *also in target space*. It resembles the real-world situation of deorphanizing receptors where information from related proteins of the ‘orphan receptor’ is taken into account to identify bioactive chemical matter for a receptor for which no ligands have been identified yet [55,56]. Moreover, this concept is applicable to predict which drug to use against a particular receptor mutant in case of *e.g*. personalized medicines, such as in case of the question which drug to use against a particular HIV genotype [7]. Since the ACE inhibitor set consisted of bioactive compounds only, LOSO could not be performed on this set.

### Compound descriptors

Ligands were described using ECFP_6 circular fingerprints calculated in Pipeline Pilot 8.5 [57], which take into account the number of connections to an atom, the element type, the charge, and the atomic mass. These descriptors have previously been shown to perform well in comparative virtual screening studies [58].This ligand side descriptor was employed for all studies presented in this work when encoding small molecule information. Here an array size of 512 bits (each bit corresponding to a chemical substructure) was used.

In addition, in the GPCR, and PI data set compounds were described by their physicochemical properties. The reason for this was that, while the NNRTI set was a congeneric series, the GPCR and PI sets were diverse. Initial trials showed that only ECFP_6 descriptors performed worse than a combination of ECFP_6 and physicochemical properties. This effect was absent in the NNRTI set. Physicochemical properties included logP, log solubility, atom properties (number of atoms, positive and negative atoms, hydrogen bond donors and acceptors), size related properties (Volume, Molecular weight, Polar Surface area), properties characterizing bonds (number of bonds, number of aromatic bonds, number of rotatable bonds), and properties describing ring and chain systems as was done previously [6]. For a full list see Supporting Additional file 1: Table S3.

### PCM modeling method

Both regression and classification models were generated in Pipeline Pilot Version 8.5 using the R-statistics modeling package version 2.12.1 [50,59]. Modeling was performed using the ‘randomForest’ package in R Statistics [60]. The size of the forest was experimentally determined to be optimal at 1500 trees (1000 in the case of the ACE inhibitors), the maximum number of descriptors to be sampled at each node was set at a fraction 0.5 of the total number. Class size equalization was turned on and a performance estimate during training was obtained using out-of-bag validation. Furthermore data points were fed into the model in a randomized order (differing between repeats of an experiment). Moreover, in the 70–30 experiments models were trained in ten fold with different randomized splits to get a more reliable performance estimate.

### Model validation

In regression models both the Root-Mean-Square Error (RMSE) and the correlation coefficient intersecting the origin (R_0_^2^) were employed [61]. For the classification models the Matthews correlation coefficient (MCC) was used to estimate model performance as it incorporates both correct and false predictions [62]. However, because of the importance of models to actually retrieve active compounds, sensitivity was employed as a second performance measure.

### Comparison to QSAR models

For the PCM datasets (GPCR, NNRTI, and PI) also dedicated QSAR models were trained per target using a 70% - 30% approach. Of these models the average RMSE / MCC and R_0_^2^ / sensitivity were calculated along with the standard deviation. The results of these models are shown in Additional file 1: Figure S18. The PCM models outperform the QSAR models (on all datasets and with all descriptors). Only in the case of the PIs the RMSE of the QSARs is slightly lower (0.23 (±0.22) log units versus 0.31 (± 0.03) log units), however the R_0_^2^ is considerably worse (0.13 (±0.28) versus 0.85 (±0.03)).

### Y-Scrambling

To make sure that the models created were not based on chance correlations, 10 fold Y-scrambling or permutation testing was performed. These studies were performed using the same setup as the benchmark experiments (also in ten fold), however the output variable (pIC_50_, pEC_50_, fold change, or activity class) was randomized over the data points. Hence no correlation should exist between the descriptors (ligand and target) and the activity when attempting to derive ‘models’. The results are shown in Additional file 1: Figures S19, S20, S21, S22 and confirm that no predictive models can be trained on this randomized set.

### Descriptor set ranking

Finally, to obtain a broadly derived performance measure all 16 amino acid descriptor sets were ranked based on their performance per dataset per experiment per validation parameter. This rank-based assessment prevents a single dataset that is modeled very well or very badly (as expressed in RMSE or MCC) unduly influencing the average performance of this descriptor set. Descriptor sets were ranked twice per experiment using 2 validation parameters (R_0_^2^ and RMSE in the case of regression and MCC and Sensitivity in the case of classification). Hence this leads to 14 ranks (ACE inhibitors R_0_^2^ rank, ACE inhibitors RMSE rank, GPCR 70–30 MCC rank, GPCR 70–30 Sensitivity rank, etc.). These validation parameters were the mean of the ten repeats of each model. Subsequently the median rank and the MAD were determined of all descriptor sets based on these 14 ranks. This lead to a final rank of each descriptor set that could be compared over all data sets.

## Consent

All patient data used in our manuscript were obtained from different collaborators. With each of these collaborators a contract was signed stipulating that patient consent was available from local IRB and/or the competent IRB/EC authorizations were obtained to provide us with the patient samples for research purposes.

## Abbreviations

AA: Amino acid; ACE: Angiotensin-converting enzyme; CV: Cross validated; FASGAI: Factor analysis scales of generalized AA information; GPCR: G protein-coupled receptor; HIV: Human immunodeficiency virus; LOSO: Leave-one-sequence-out; MCC: Matthews correlation coefficient; NNRTI: Non-nucleoside reverse transcriptase inhibitor; PC: Principal component; PCA: Principal component analysis; PCM: Proteochemometric; PI: Protease inibitor; ProtFP: Protein fingerprint; QSAM: Quantitative sequence-cctivity Modeling; QSAR: Quantitative structure-activity Relationship; R02: Correlation coefficient intersecting the origin (0,0); RMSE: Root-mean-square error; SEM: Standard error of the mean; Sens: Sensitivity; TLC: Thin layer chromatography; TM: Trans-membrane; VHSE: Vectors of hydrophobic,sSteric, and electronic properties.

## Competing interests

The authors declare no competing interests.

## Authors’ contributions

GJPvW conceived the study, participated in its design, carried out calculations, and drafted the manuscript. RFS carried out calculations, and helped draft the manuscript. IC participated in data analysis, created figures and helped draft the manuscript. JKW participated in study design. JPO superimposed the crystal structures and selected binding site residues. APIJ helped draft the manuscript. HWTvV participated in study design and drafted the manuscript. AB drafted the manuscript and participated in study design. All authors read and approved the final manuscript.

## Supplementary Material

Additional file 1: Table S1 Model training values ACE inhibitor set. **Table S2.** Receptors used in the GPCR set. **Table S3.** Physicochemical classifiers used as compound descriptors. **Figure S1.** PCA analysis of the 58 ACE inhibiting peptides (I). **Figure S2.** PCA analysis of the 58 ACE inhibiting peptides (II). **Figure S3.** PCA analysis of the 58 ACE inhibiting peptides (III). **Figure S4.** GPCRs in 70-30 validation. **Figure S5.** GPCRs in LOSO validation. **Figure S6.** PCA analysis of the GPCR target space (I). **Figure S7.** PCA analysis of the GPCR target space (II). **Figure S8.** PCA analysis of the GPCR target space (III). **Figure S9.** RT mutants in 70-30 validation. **Figure S10.** RT mutants in LOSO validation. **Figure S11.** PCA analysis of the NNRTI target space (I). **Figure S12.** PCA analysis of the NNRTI target space (II). **Figure S13.** PCA analysis of the NNRTI target space (III). **Figure S14.** PCA analysis of the PI target space (I). **Figure S15.** PCA analysis of the PI target space (II). **Figure S16.** PCA analysis of the PI target space (III). **Figure S17.** The GPCR Set. **Figure S18.** QSAR experiments. **Figure S19.** ACE inhibitor 10 fold y-scrambling. **Figure S20.** GPCR 10 fold y-scrambling. **Figure S21.** NNRTI 10-fold y-scrambling. **Figure S22.** PI 10-fold y-scrambling. Click here for file

Additional file 2 Furthermore, we include a Pipeline Pilot component to convert single letter AA sequences to any of the here tested descriptor sets and a fully functional example protocol, both to be used in Pipeline Pilot 8.5 and up. Click here for file

Additional file 3 **The multiple sequence alignment used for the GPCR binding site description (includes: residue types, Ballesteros-Weinstein numbers, GPCRdb numbers, and receptor specific residue numbers).** The GPCR and ACE data sets are available for download at http://www.gjpvanwesten.nl/proteindescriptors but were considered too large to submit with the paper. Click here for file
