# Supplementary material for: Benchmarking of protein descriptor sets in proteochemometric modeling (part 2): modeling performance of 13 amino acid descriptor sets
Source: J Cheminform. 2013 Sep 24;5:42. doi: 10.1186/1758-2946-5-42 (PMC4015169; doi:10.1186/1758-2946-5-42)
Supplement: Additional file 1: Table S1 — Model training values ACE inhibitor set. Table S2. Receptors used in the GPCR set. Table S3. Physicochemical classifiers used as compound descriptors. Figure S1. PCA analysis of the 58 ACE inhibiting peptides (I). Figure S2. PCA analysis of the 58 ACE inhibiting peptides (II). Figure S3. PCA analysis of the 58 ACE inhibiting peptides (III). Figure S4. GPCRs in 70-30 validation. Figure S5. GPCRs in LOSO validation. Figure S6. PCA analysis of the GPCR target space (I). Figure S7. PCA analysis of the GPCR target space (II). Figure S8. PCA analysis of the GPCR target space (III). Figure S9. RT mutants in 70-30 validation. Figure S10. RT mutants in LOSO validation. Figure S11. PCA analysis of the NNRTI target space (I). Figure S12. PCA analysis of the NNRTI target space (II). Figure S13. PCA analysis of the NNRTI target space (III). Figure S14. PCA analysis of the PI target space (I). Figure S15. PCA analysis of the PI target space (II). Figure S16. PCA analysis of the PI target space (III). Figure S17. The GPCR Set. Figure S18. QSAR experiments. Figure S19. ACE inhibitor 10 fold y-scrambling. Figure S20. GPCR 10 fold y-scrambling. Figure S21. NNRTI 10-fold y-scrambling. Figure S22. PI 10-fold y-scrambling. [file 1758-2946-5-42-S1.pdf]

# Benchmarking of Protein Descriptor Sets in Proteochemometric Modeling (Part 2): Modeling Performance of 13 Amino Acid Descriptor Sets

**Additional File 1**

*Gerard J.P. van Westen [1*], Remco F. Swier [1], Isidro Cortes-Ciriano[2], Jörg K. Wegner[3], John P. Overington[4], Adriaan P. IJzerman [1], Herman W.T. van Vlijmen [1, 3], and Andreas Bender [1,5]*

[1] Division of Medicinal Chemistry, Leiden / Amsterdam Center for Drug Research, Einsteinweg 55, 2333 CC, Leiden, The Netherlands

[2] Unité de Bioinformatique Structurale, Institut Pasteur and CNRS URA 2185, Structural Biology and Chemistry Department, 25-28, rue du Dr. Roux, 75 724 Paris, France

[3] Tibotec BVBA, Turnhoutseweg 30, 2340 Beerse, Belgium

[4] ChEMBL Group, European Molecular Biology Laboratory European Bioinformatics Institute (EMBL-EBI), Wellcome Trust Genome Campus, CB10 1SD, Hinxton, United Kingdom

[5] Unilever Centre for Molecular Science Informatics, Department of Chemistry, University of Cambridge, Lensfield Road, Cambridge, CB2 1EW, United Kingdom

**Table of Contents:**

Supporting Table S1. Model training values ACE inhibitor set. ....................................... 3
Supporting Table S2. Receptors used in the GPCR set. ................................................... 4
Supporting Table S3. Physicochemical classifiers used as compound descriptors. ........... 5
Supporting figure S1. PCA analysis of the 58 ACE inhibiting peptides (I) ...................... 6
Supporting figure S2. PCA analysis of the 58 ACE inhibiting peptides (II). .................... 7
Supporting figure S3. PCA analysis of the 58 ACE inhibiting peptides (III).................... 8
Supporting figure S4. GPCRs in 70-30 validation............................................................ 9
Supporting figure S5. GPCRs in LOSO valiation. ......................................................... 10
Supporting figure S6. PCA analysis of the GPCR target space (I)................................. 11
Supporting figure S7. PCA analysis of the GPCR target space (II). ............................... 12
Supporting figure S8. PCA analysis of the GPCR target space (III). .............................. 13
Supporting figure S9. RT mutants in 70-30 validation.................................................... 14
Supporting figure S10 RT mutants in LOSO validation.................................................. 15
Supporting figure S11. PCA analysis of the NNRTI target space (I). ............................. 16
Supporting figure S12. PCA analysis of the NNRTI target space (II)............................. 17
Supporting figure S13. PCA analysis of the NNRTI target space (III). .......................... 18
Supporting figure S14. PCA analysis of the PI target space (I)....................................... 19
Supporting figure S15. PCA analysis of the PI target space (II). .................................... 20
Supporting figure S16. PCA analysis of the PI target space (III). ................................... 21
Supporting Figure S17. The GPCR Set ........................................................................... 22
Supporting figure S18. QSAR experiments..................................................................... 23
Supporting figure S19. ACE inhibitor 10 fold y-scrambling........................................... 24
Supporting figure S20. GPCR 10 fold y-scrambling....................................................... 25
Supporting figure S21. NNRTI 10-fold y-scrambling..................................................... 26
Supporting figure S22. PI 10-fold y-scrambling............................................................. 27

**Supporting Table S1. Model training values ACE inhibitor set.**

| Descriptor Set | CV RMSE | $Q^2$ | Published $R^2$ | Published $Q^2$ | Published RMSE |
|---|---|---|---|---|---|
| BLOSUM | 0.52 (±0.06) | 0.74 (±0.09) | n/a | n/a | n/a |
| FASGAI | 0.47 (±0.04) | 0.80 (±0.05) | 0.76 | 0.73 | 0.50 |
| MSWHIM | 0.49 (±0.04) | 0.77 (±0.06) | 0.71 | 0.64 | 0.54 |
| ProtFP (PCA3) | 0.52 (±0.04) | 0.76 (±0.05) | n/a | n/a | n/a |
| ProtFP (PCA5) | 0.53 (±0.04) | 0.73 (±0.07) | n/a | n/a | n/a |
| ProtFP (PCA8) | 0.53 (±0.05) | 0.73 (±0.09) | n/a | n/a | n/a |
| ProtFP (Feature) | 0.69 (±0.06) | 0.52 (±0.10) | n/a | n/a | n/a |
| ST-scales | 0.52 (±0.05) | 0.74 (±0.08) | 0.86 | 0.77 | 0.40 |
| T-scales | 0.47 (±0.04) | 0.78 (±0.06) | 0.85 | 0.79 | 0.46 |
| VHSE | 0.48 (±0.04) | 0.78 (±0.06) | 0.77 | 0.75 | 0.48 |
| Z-Scales (3) | 0.47 (±0.04) | 0.80 (±0.06) | 0.77 | 0.72 | n/a |
| Z-scales (5) | 0.46 (±0.04) | 0.80 (±0.05) | n/a | n/a | n/a |
| Z-scales (Binned) | 0.46 (±0.04) | 0.80 (±0.05) | n/a | n/a | n/a |
| Z-Scales (3) and ProtFP (Feature) | 0.49 (±0.04) | 0.78 (±0.07) | n/a | n/a | n/a |
| Z-Scales (3) and Z-Scales (Avg) | 0.51 (±0.04) | 0.76 (±0.06) | n/a | n/a | n/a |
| Z-Scales (Binned) and ProtFP (PCA3) | 0.47 (±0.03) | 0.79 (±0.05) | n/a | n/a | n/a |

Experiments were performed 10 times and the stddev is given in parentheses. Also shown are the published values obtained from literature if available. CV RMSE is cross validated RMSE

**Supporting Table S2. Receptors used in the GPCR set.**

| Receptor | Family | Actives | Inactives |
|---|---|---|---|
| 5HT1A | Serotonin Receptor | 100 | 100 |
| 5HT1B | | 100 | 99 |
| 5HT1D | | 100 | 90 |
| 5HT2A | | 100 | 100 |
| 5HT2B | | 100 | 100 |
| 5HT2C | | 100 | 100 |
| 5HT4 | | 100 | 75 |
| 5HT5A | | 35 | 78 |
| 5HT6 | | 100 | 100 |
| 5HT7 | | 100 | 100 |
| ACM1 | Muscarinic Acetylcholine Receptor | 100 | 100 |
| ACM2 | | 100 | 100 |
| ACM3 | | 100 | 100 |
| ACM4 | | 79 | 100 |
| ACM5 | | 46 | 100 |
| ADA1A | Alpha Adrenergic Receptor | 100 | 100 |
| ADA1B | | 100 | 100 |
| ADA1D | | 100 | 100 |
| ADA2A | | 100 | 100 |
| ADA2B | | 76 | 100 |
| ADA2C | | 100 | 100 |
| ADRB1 | Beta Adrenergic Receptor | 71 | 100 |
| ADRB2 | | 100 | 100 |
| ADRB3 | | 66 | 100 |
| DRD1 | Dopamine Receptor | 100 | 100 |
| DRD2 | | 100 | 100 |
| DRD3 | | 100 | 100 |
| DRD4 | | 100 | 100 |
| DRD5 | | 53 | 78 |
| HRH1 | Histamine Receptor | 100 | 100 |
| HRH3 | | 100 | 100 |
| HRH4 | | 100 | 100 |

**Supporting Table S3. Physicochemical classifiers used as compound descriptors.**

| Descriptor | Binvalue | | | |
|---|---|---|---|---|
| | -- | - | + | ++ |
| LogD | < - 0.50 | >= -0.50 & < 3.40 | >= 3.40 & =< 7.50 | > 7.50 |
| Molecular Solubility | < -9 | >= -9 & < -6.4 | >= -6.4 & =< -4 | > -4 |
| | | | | |
| Number of Atoms | < 20 | n/a | >= 20 & =< 40 | > 40 |
| Number of Hydrogens | < 16 | >= 16 & < 24 | >= 24 & =< 40 | > 40 |
| Positive Atoms | < 1 | n/a | >= 1 & =< 2 | > 2 |
| Negative Atoms | < 1 | n/a | >= 1 & =< 2 | > 2 |
| Hydrogenbond Acceptors | < 3 | >= 3 & < 5 | >= 5 & =< 8 | > 8 |
| Hydrogenbond Donors | < 2 | 2 | >= 3 & =< 4 | > 4 |
| | | | | |
| Molecular Weight | < 300 | >= 300 & < 500 | >= 500 & =< 650 | > 650 |
| Molecular Surface Area | < 200 | >= 200 & < 350 | >= 350 & =< 550 | > 550 |
| Polar Surface Area | < 100 | >= 100 & < 250 | >= 250 & =< 500 | > 500 |
| Molecular Volume | < 200 | >= 200 & <400 | >= 400 & =< 700 | > 700 |
| | | | | |
| Number of Bonds | < 20 | >= 20 & < 30 | >= 30 & =< 50 | > 50 |
| Number of Ringbonds | < 7 | >= 7 & < 18 | >= 18 & =< 32 | > 32 |
| Number of Aromatic Bonds | < 7 | >= 7 & < 12 | >= 12 & =< 18 | > 18 |
| Number of Bridgebonds | < 1 | n/a | >= 1 & =< 8 | > 8 |
| Number of Rotatable Bonds | < 5 | >= 5 & < 7 | >= 7 & =< 10 | > 10 |
| | | | | |
| Number of Rings | < 4 | 4 | >= 4 & =< 6 | > 6 |
| Number of Chains | < 5 | >= 5 & < 7 | >= 7 & =< 11 | > 11 |
| Number of Ring Assemblies | < 3 | >= 3 & < 4 | >= 4 & =< 6 | > 6 |
| Number of Chain Assemblies | < 3 | >= 3 & < 4 | >= 4 & =< 7 | > 7 |
| Number of Aromatic Rings | < 3 | >= 3 & < 4 | >= 4 & =< 6 | > 6 |

The chemical descriptors were binned after their distribution in the data set was studied. In four cases only three bins were used as the distribution would lead to sparsely filled bins, here - was omitted.

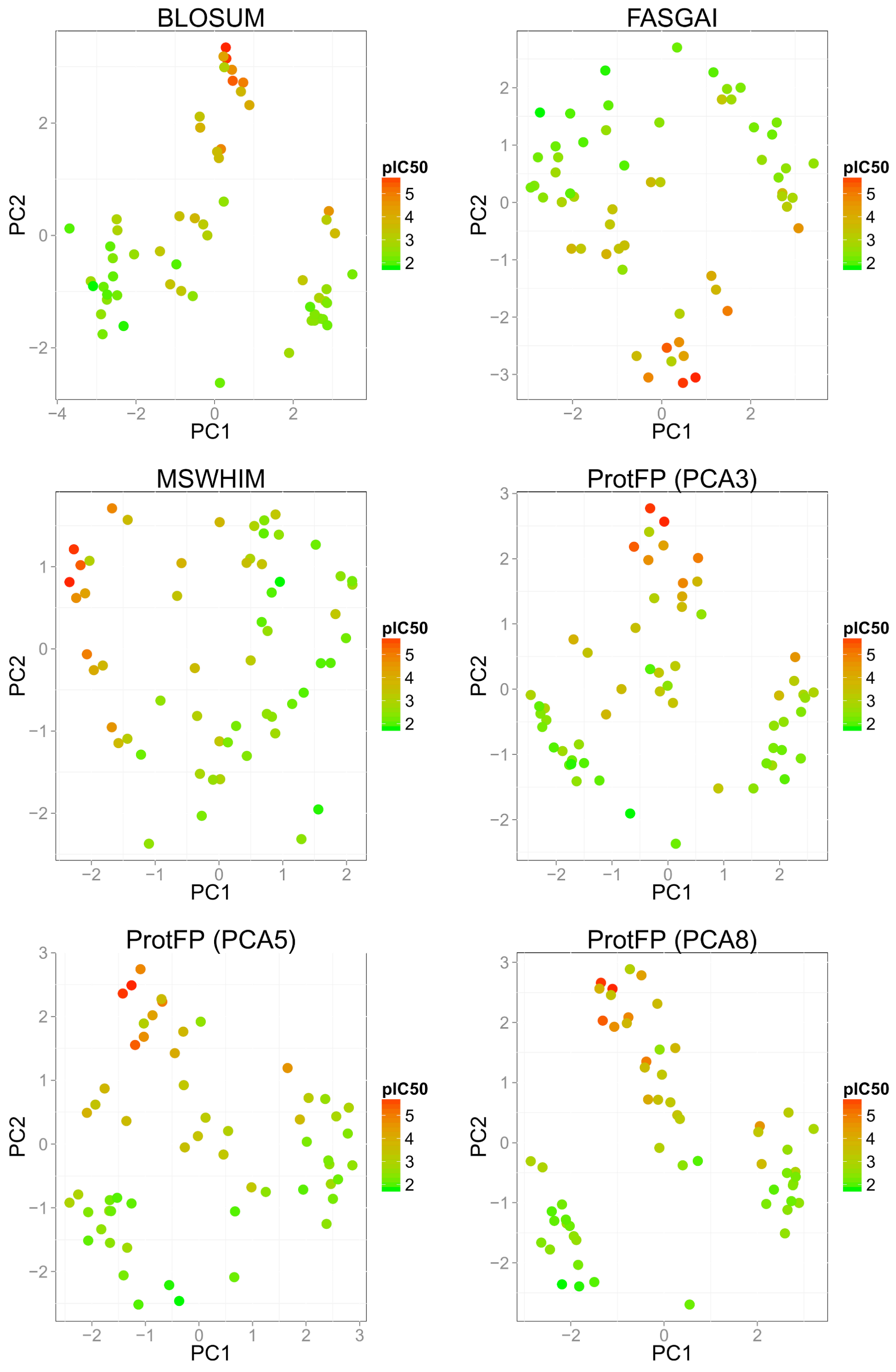


**Supporting figure S1.** PCA analysis of the 58 ACE inhibiting peptides (I)**.** Data points are colored by activity (green pKi = 2 and red pKi = 6).

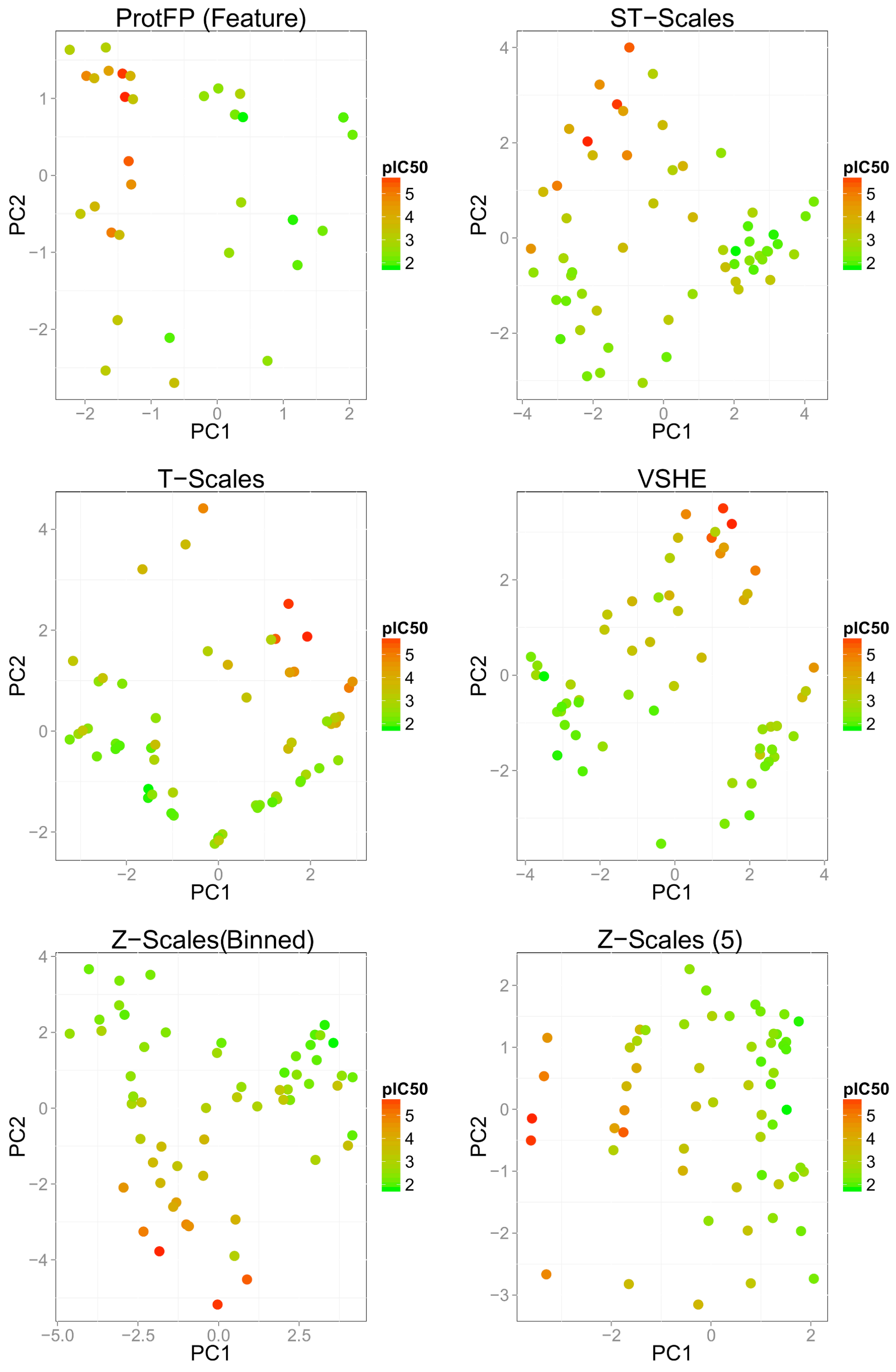


**Supporting figure S2.** PCA analysis of the 58 ACE inhibiting peptides (II). Data points are colored by activity (green pKi = 2 and red pKi = 6).

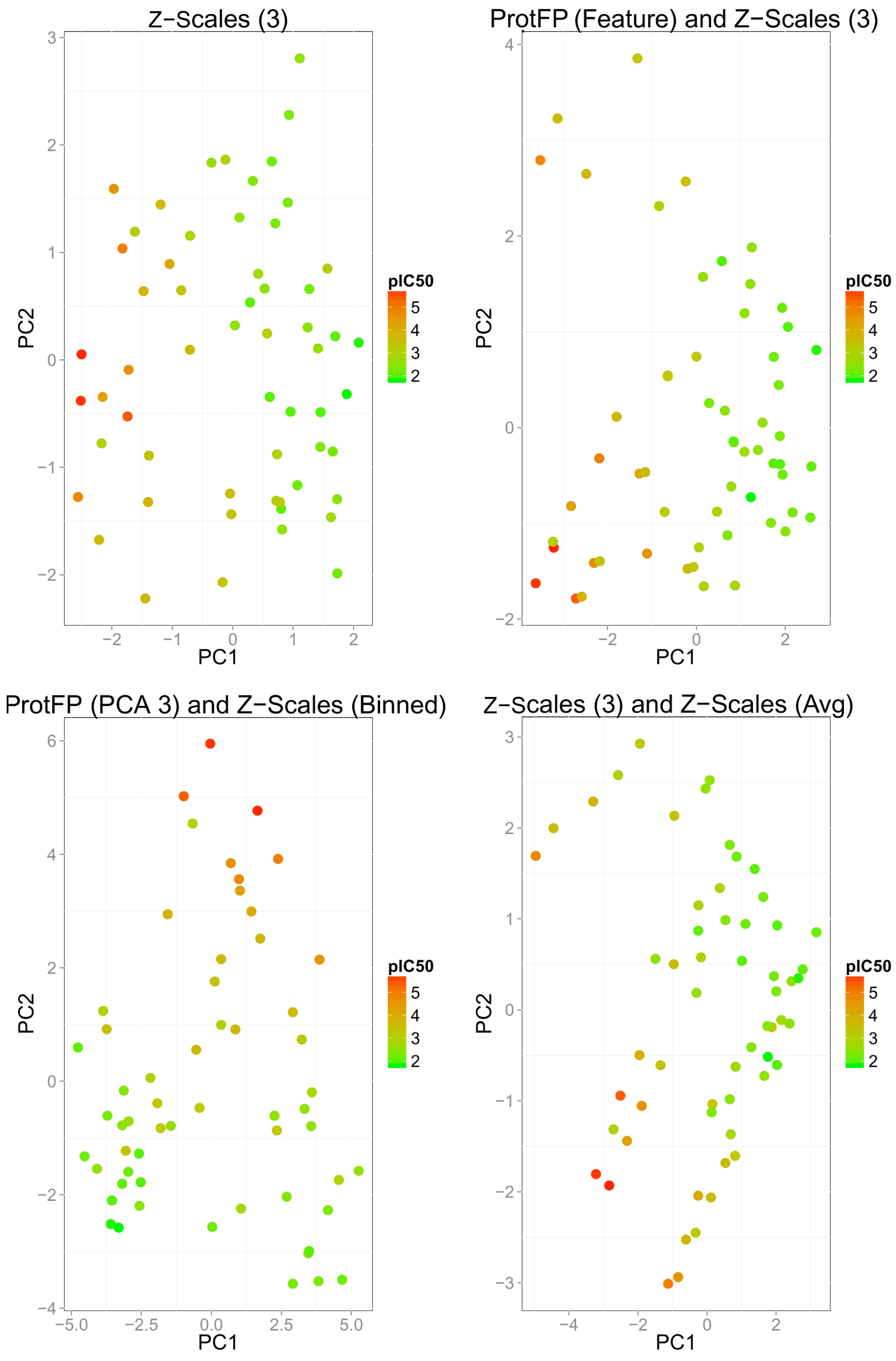


**Supporting figure S3.** PCA analysis of the 58 ACE inhibiting peptides (III). Data points are colored by activity (green pKi = 2 and red pKi = 6).

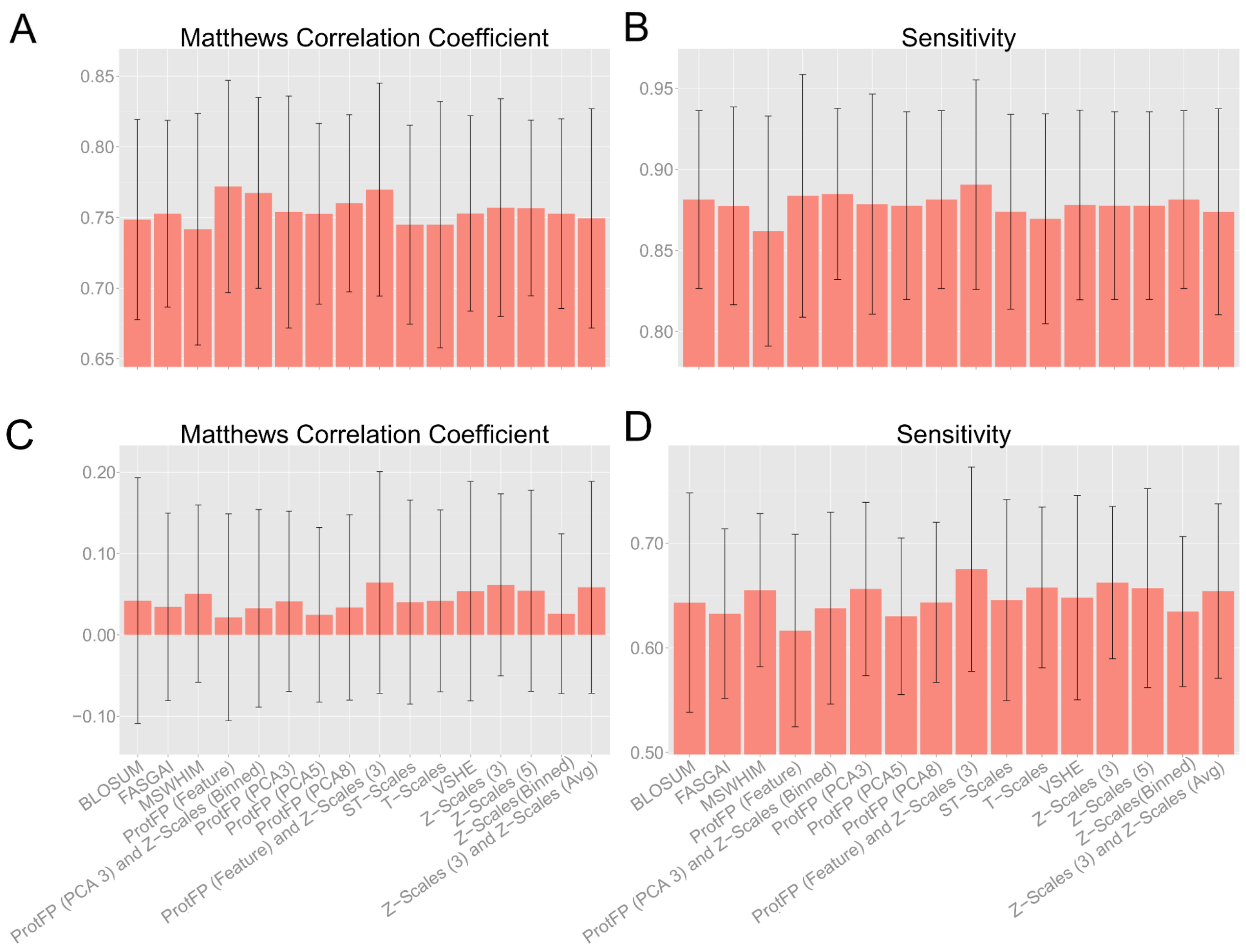


**Supporting figure S4.** GPCRs in 70-30 validation. Best performing (ACM4; A, B) and worst performing (histamine H3; C, D) GPCRs in the 70-30 validation.

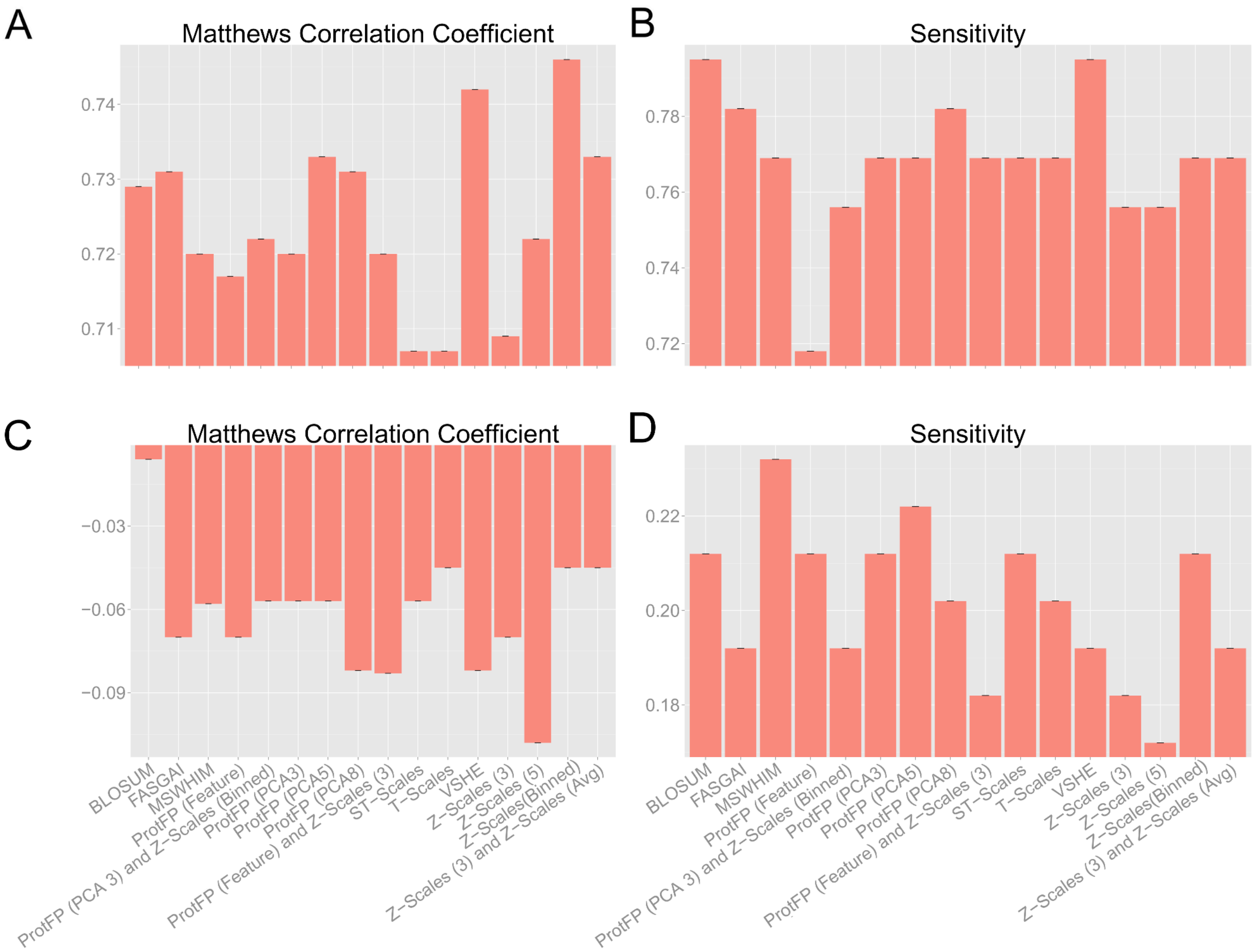


**Supporting figure S5.** GPCRs in LOSO valiation. Best performing (ACM 4; A, B) and worst performing (histamine H4; C, D) GPCRs in the LOSO validation.

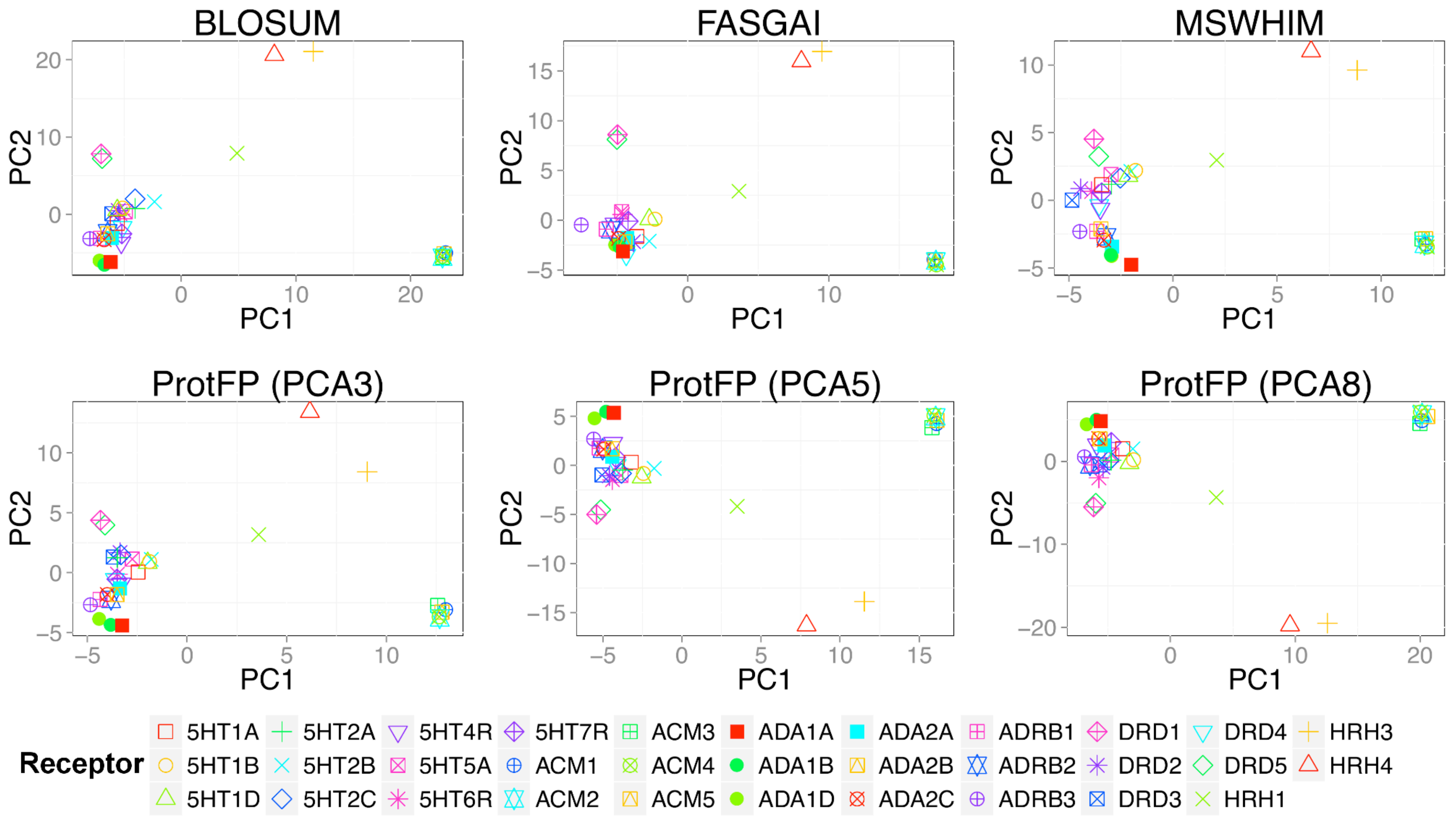


**Supporting figure S6.** PCA analysis of the GPCR target space (I). Data points are colored by receptor subfamily.

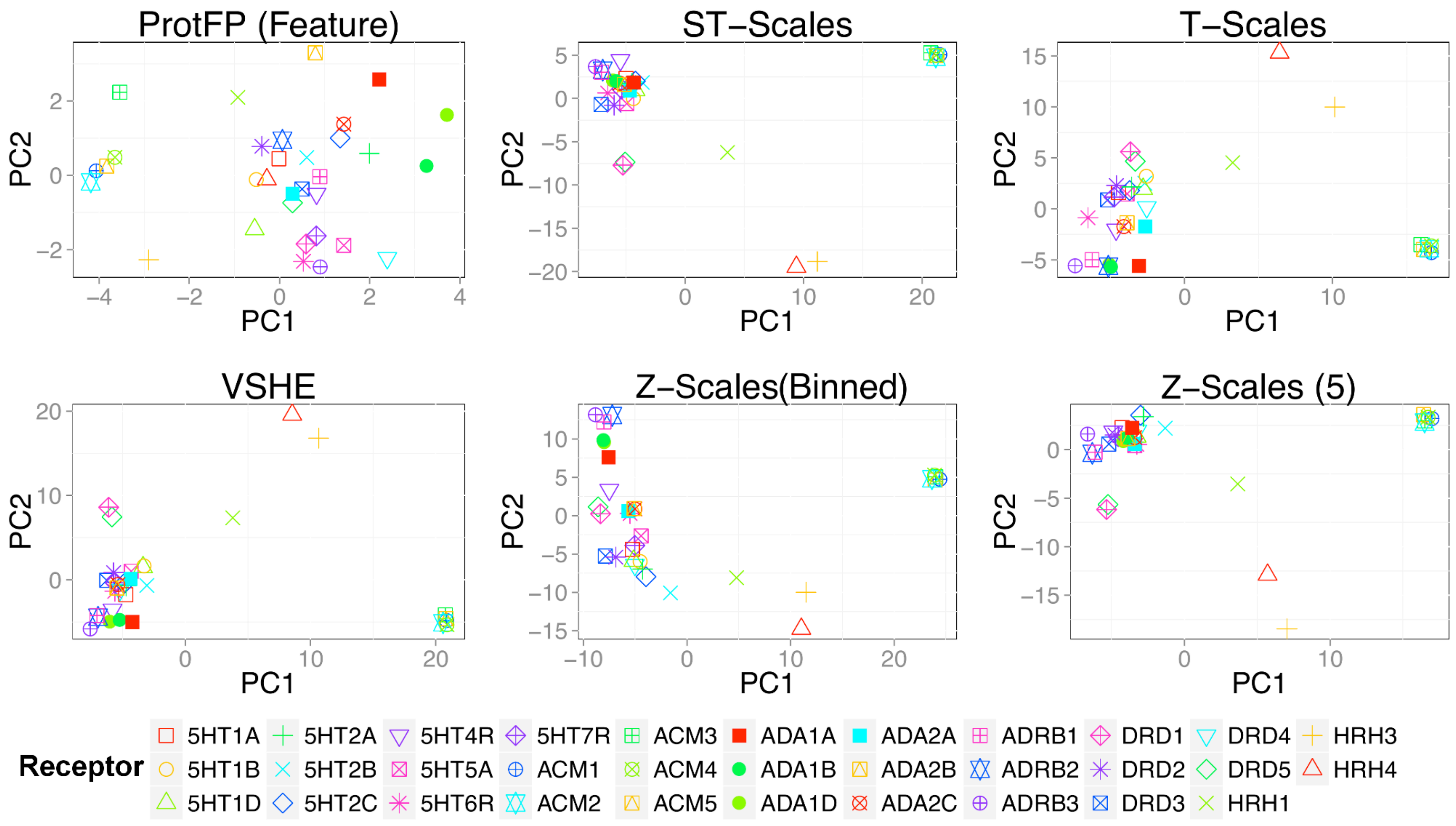


**Supporting figure S7.** PCA analysis of the GPCR target space (II). Data points are colored by receptor subfamily.

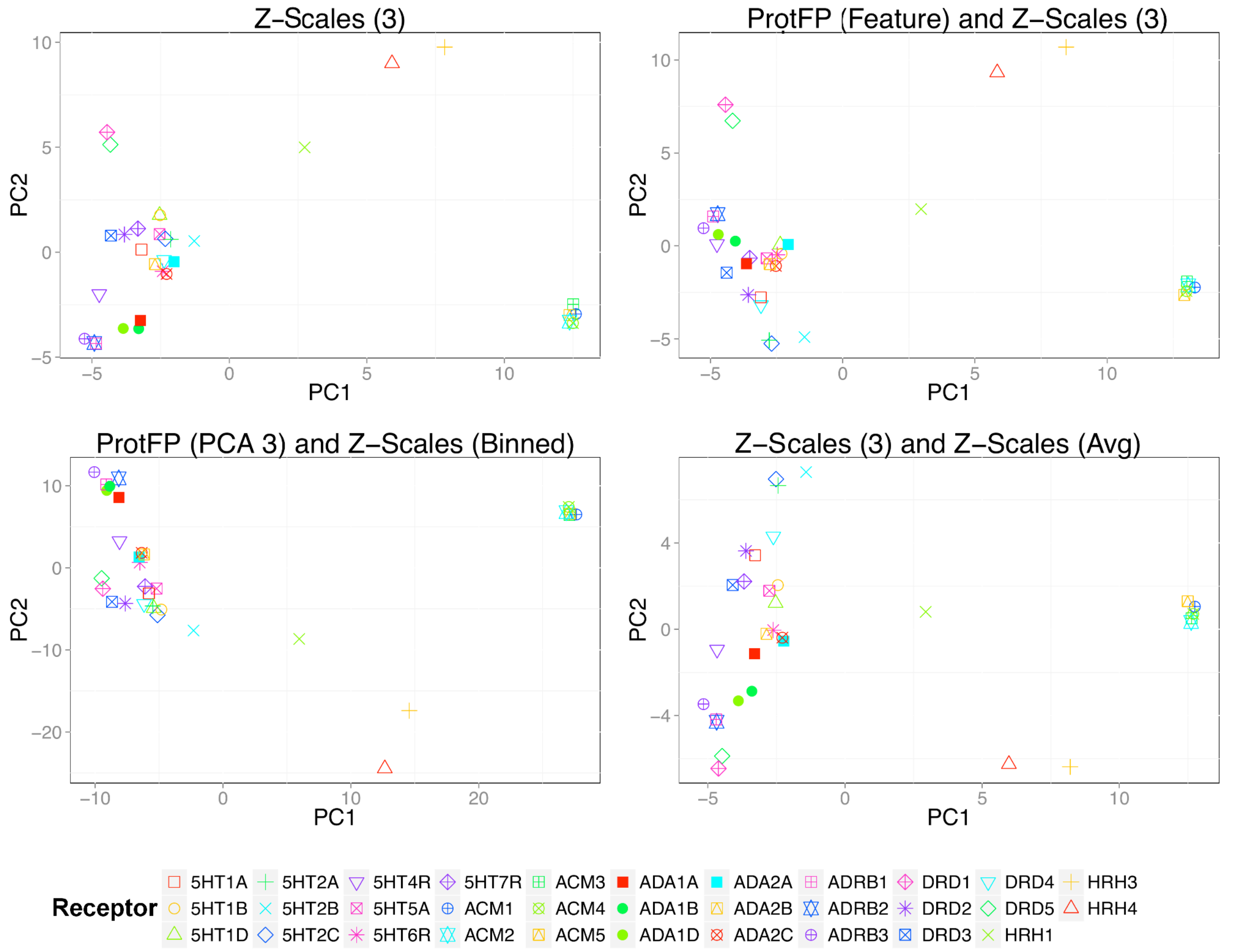


**Supporting figure S8.** PCA analysis of the GPCR target space (III). Data points are colored by receptor subfamily.

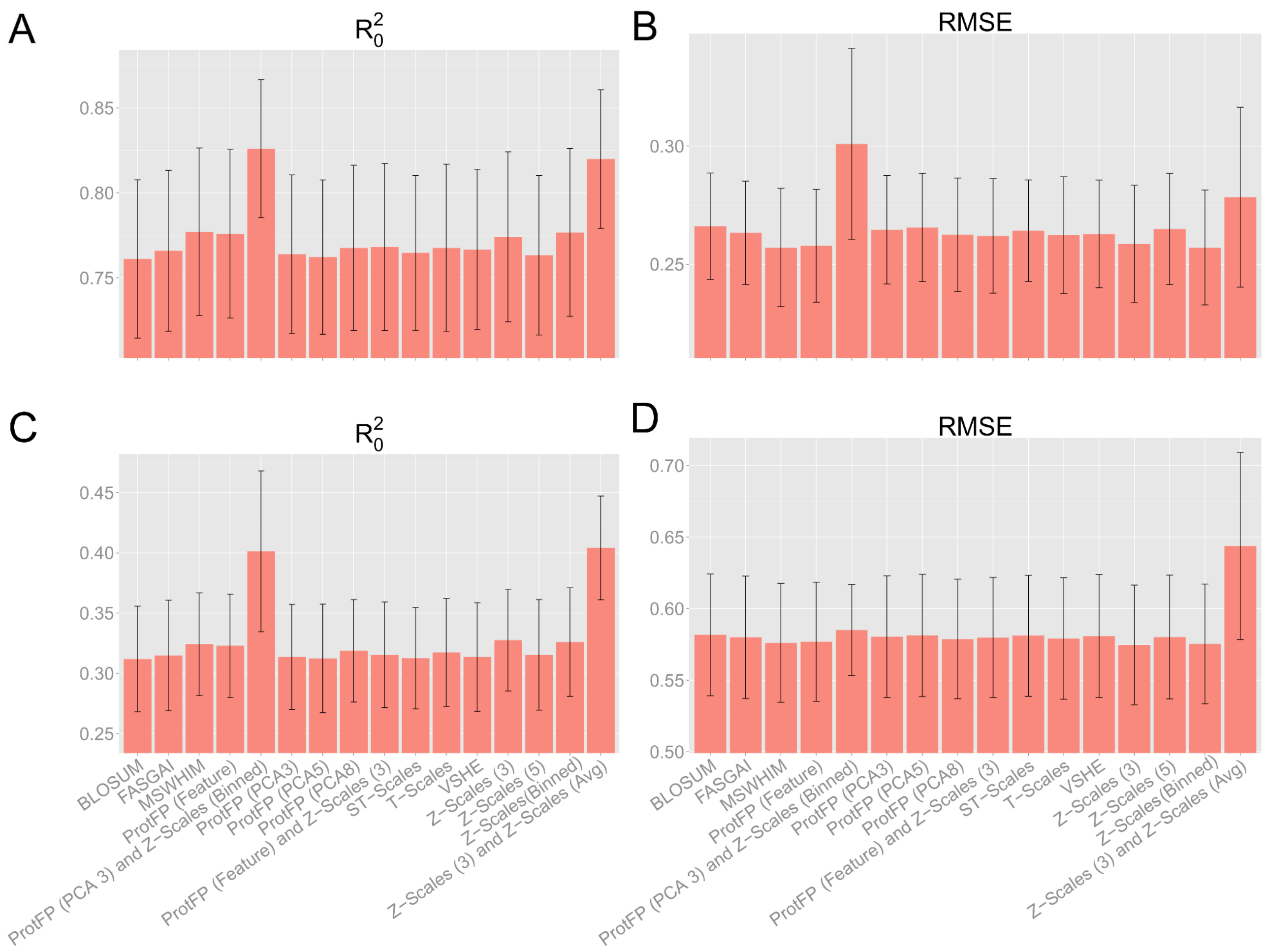


**Supporting figure S9.** RT mutants in 70-30 validation. Best performing (Sequence 12 and 9; A, B) and worst performing (Sequence 2 and 6; C, D) mutants in the 70-30 validation.

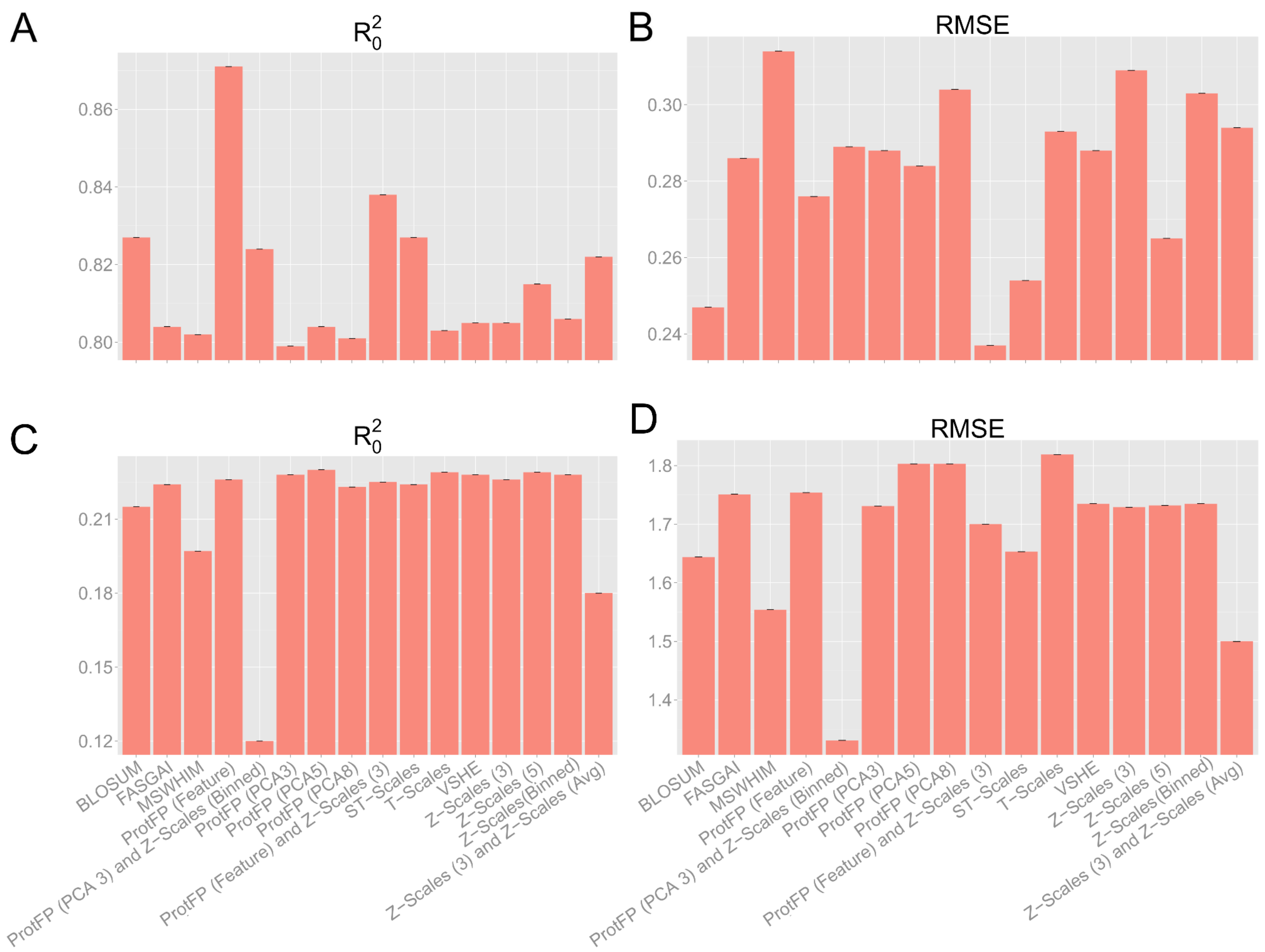


**Supporting figure S10** RT mutants in LOSO validation. Best performing (sequence 3/12; A, B) and worst performing (sequence 6; C, D) mutants in the LOSO validation.

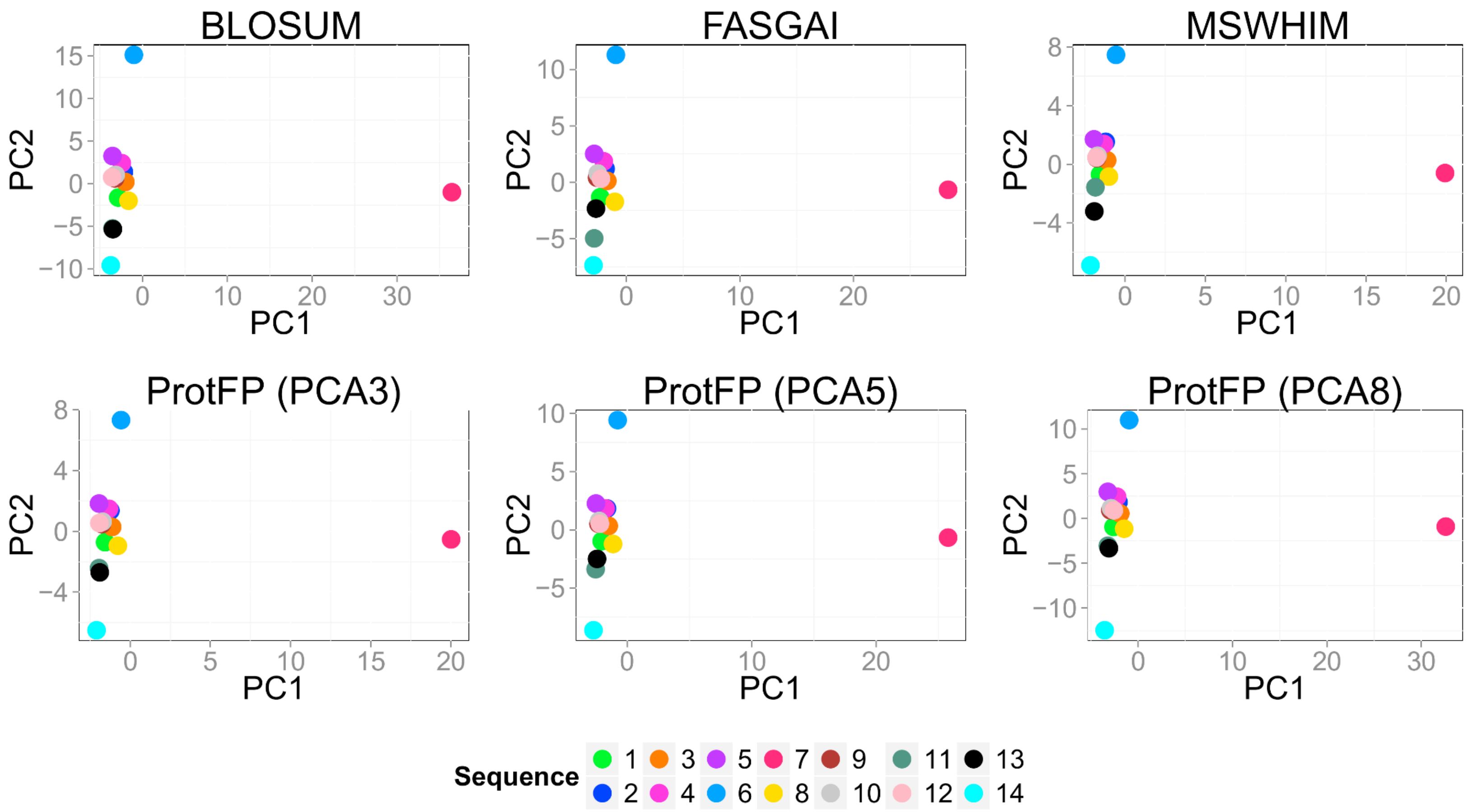


**Supporting figure S11.** PCA analysis of the NNRTI target space (I). Data points are colored by mutant.

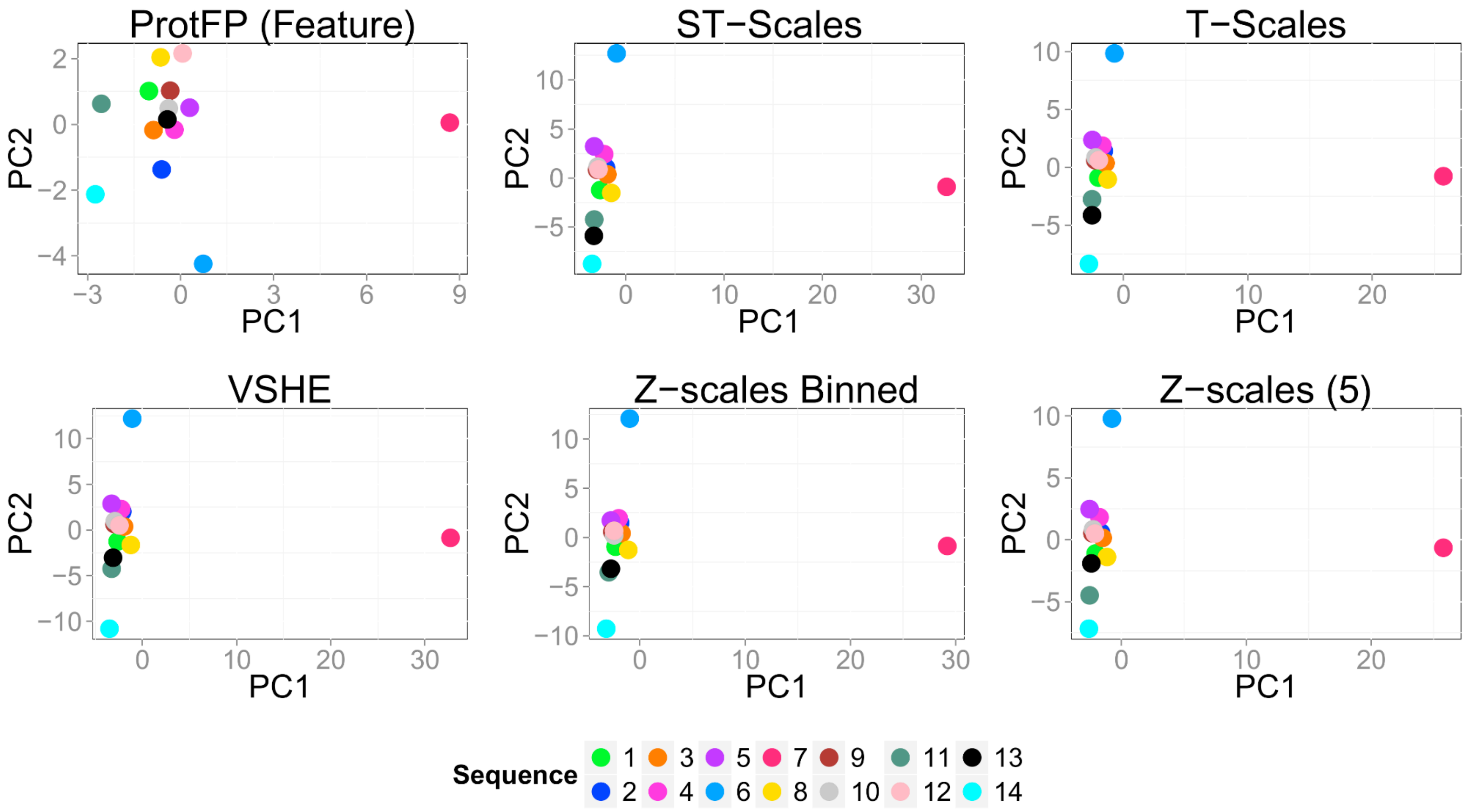


**Supporting figure S12.** PCA analysis of the NNRTI target space (II). Data points are colored by mutant.

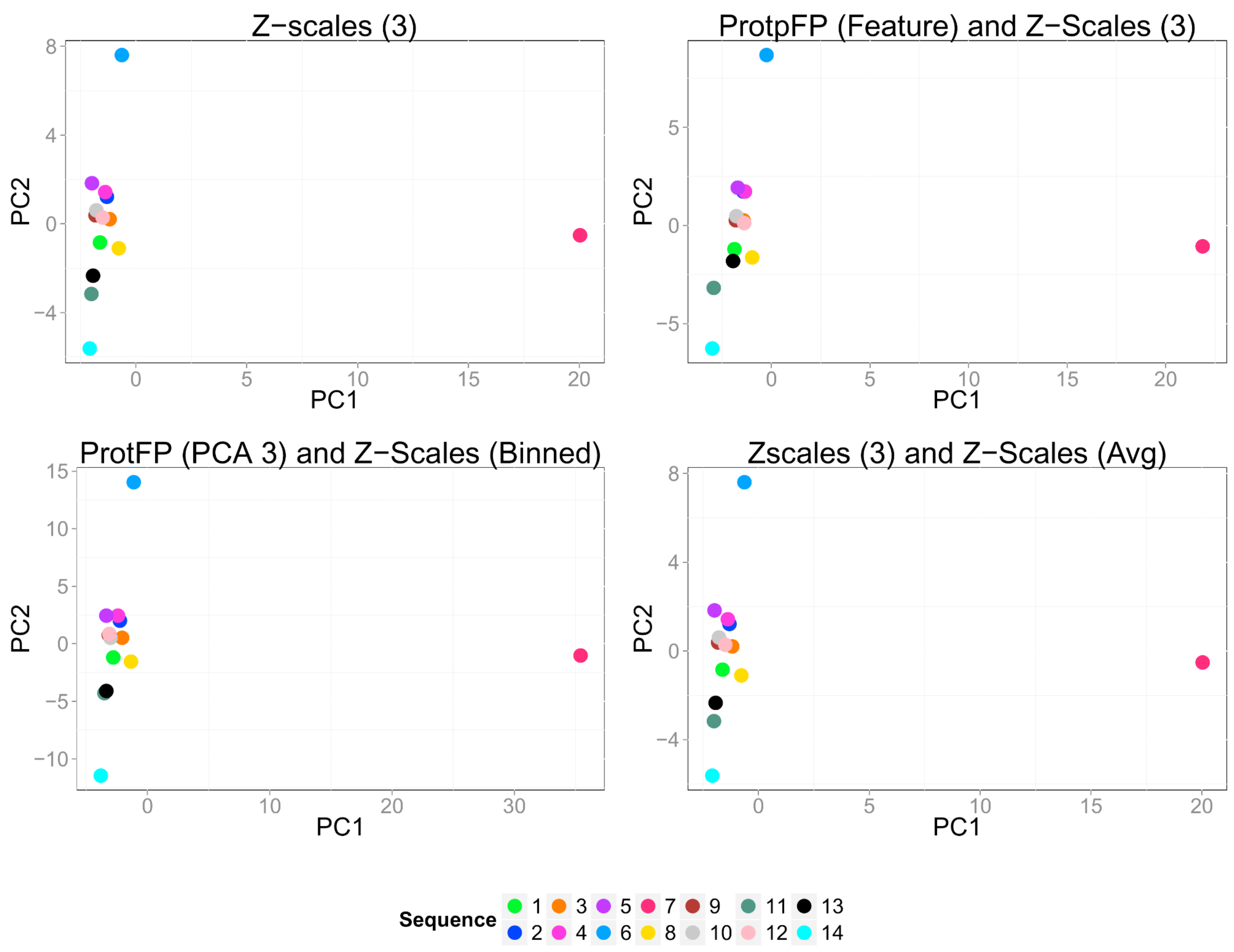


**Supporting figure S13.** PCA analysis of the NNRTI target space (III). Data points are colored by mutant.

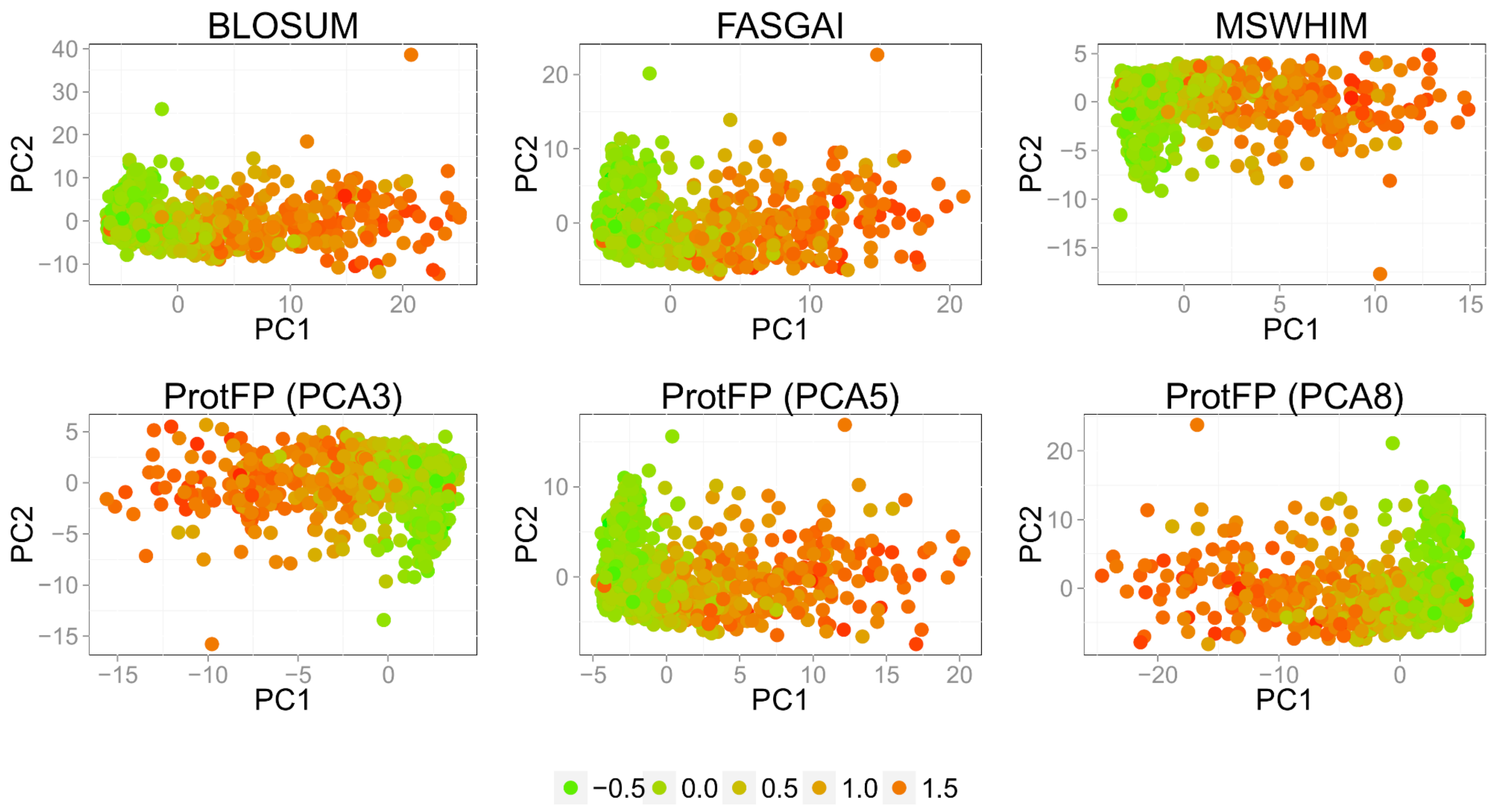


**Supporting figure S14.** PCA analysis of the PI target space (I). Data points are colored by average resistance for a mutant.

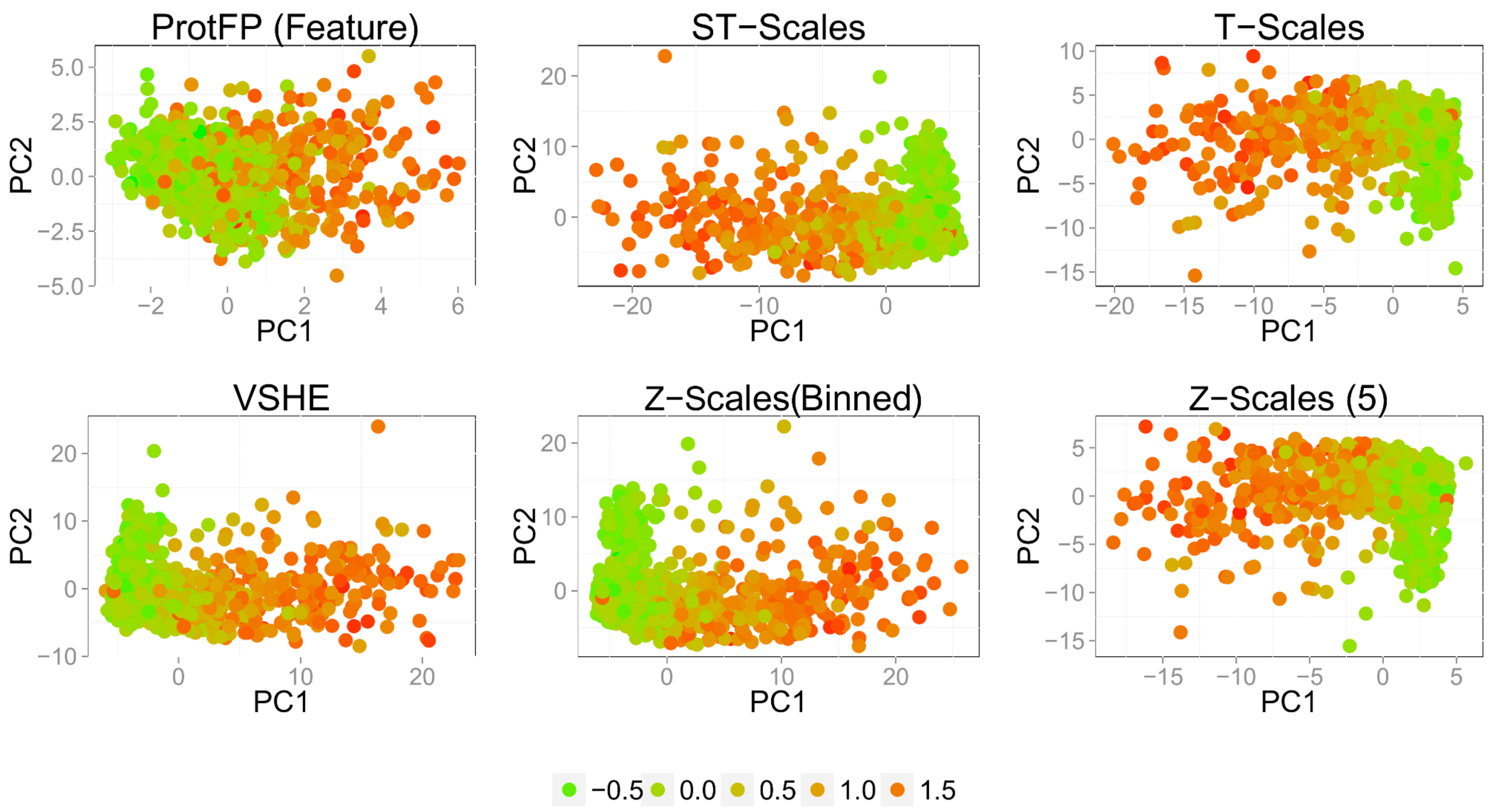


**Supporting figure S15.** PCA analysis of the PI target space (II). Data points are colored by average resistance for a mutant.

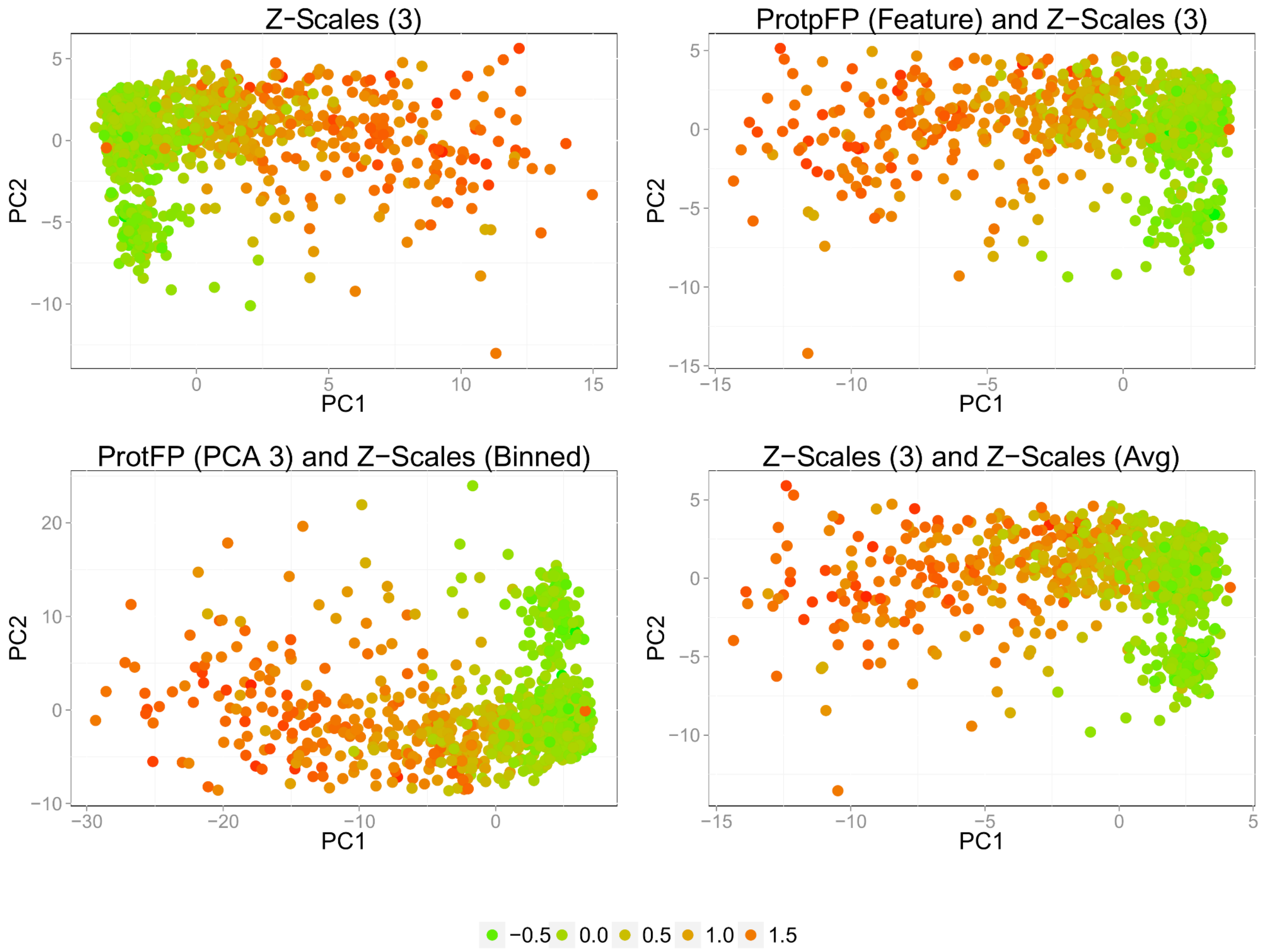


**Supporting figure S16.** PCA analysis of the PI target space (III). Data points are colored by average resistance for a mutant.

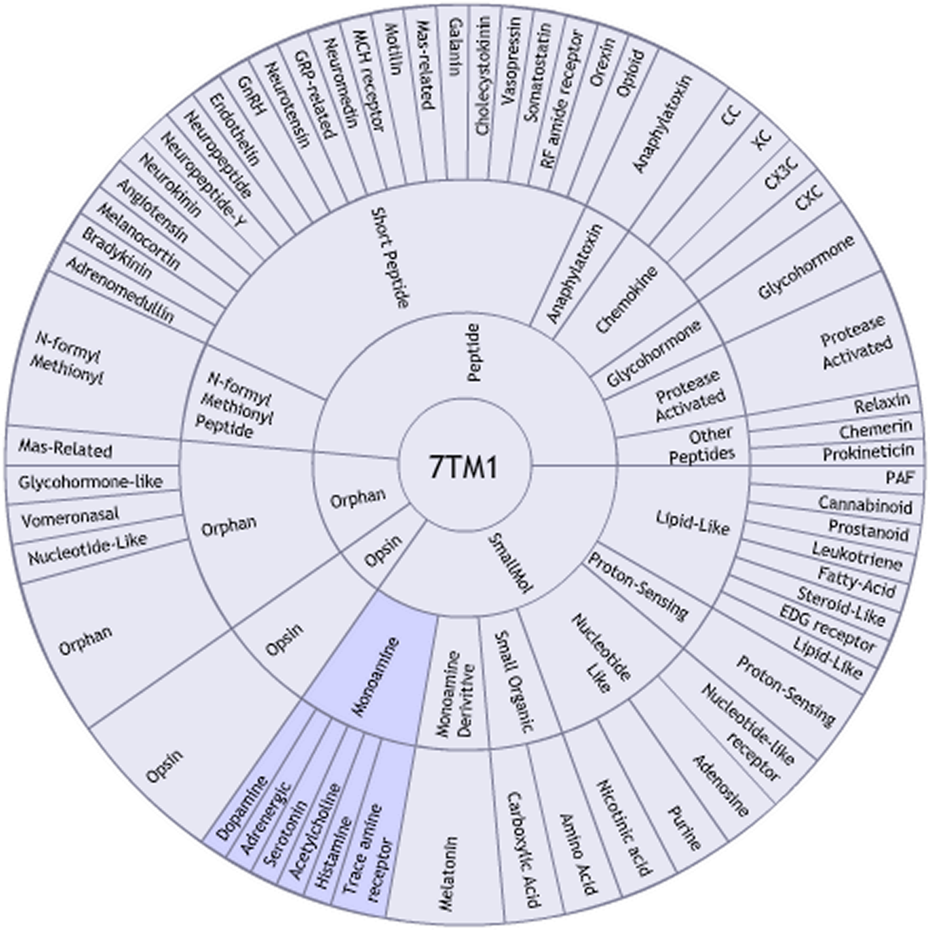


**Supporting Figure S17.** The GPCR Set was constructed on the monoamine receptor family (highlighted).

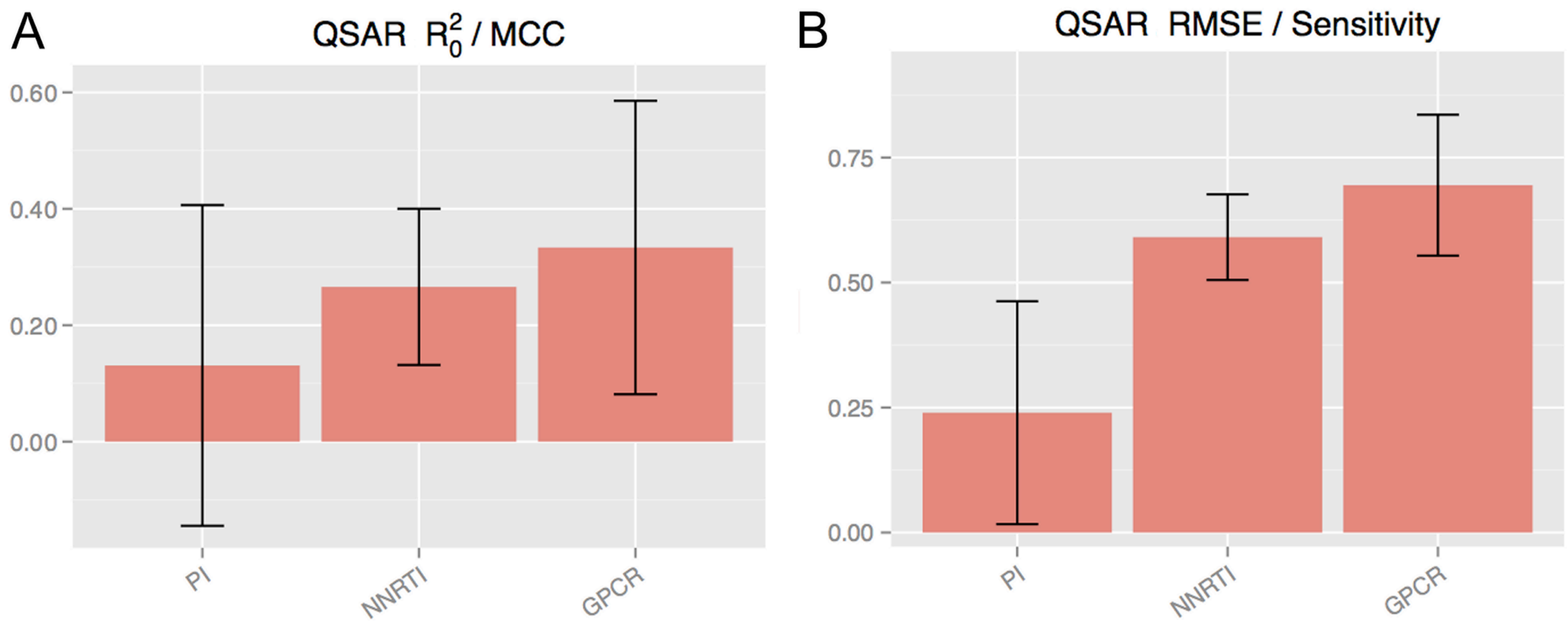


**Supporting figure S18.** QSAR experiments. Performance of dedicated QSAR models trained on the different descriptor sets.

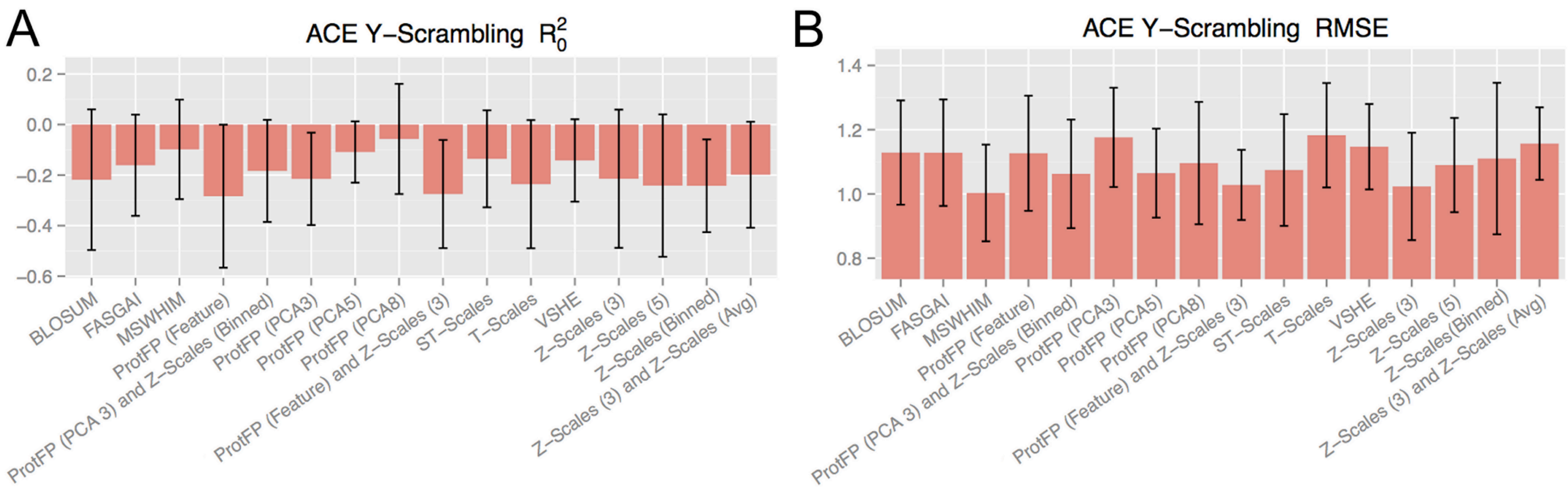


**Supporting figure S19.** ACE inhibitor 10 fold y-scrambling. Performance of the different descriptor sets on y-scrambled 70-30 ACE inhibitor set.

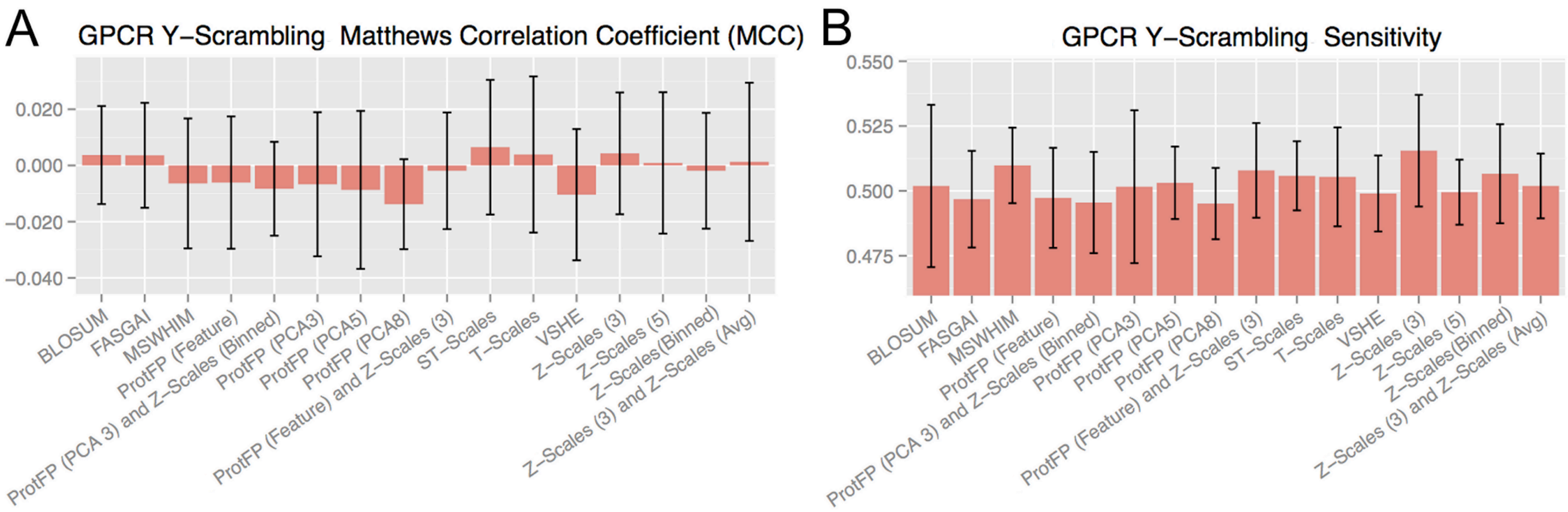


**Supporting figure S20.** GPCR 10 fold y-scrambling. Performance of the different descriptor sets on y-scrambled GPCR set.

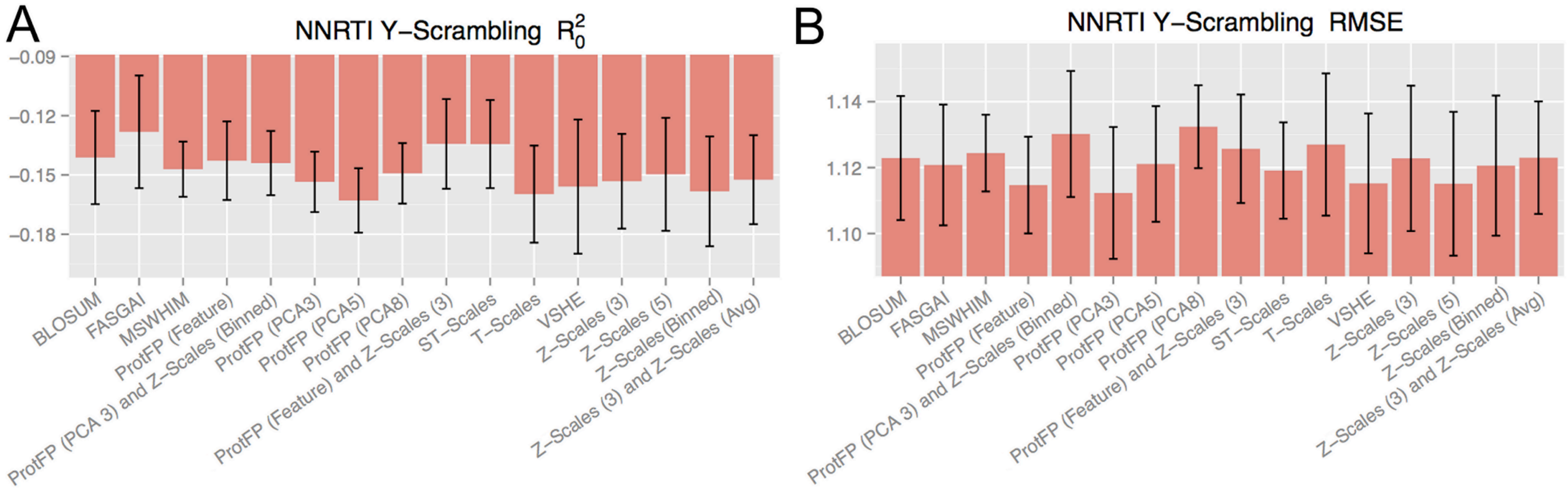


**Supporting figure S21.** NNRTI 10-fold y-scrambling. Performance of the different descriptor sets on y-scrambled NNRTI set.

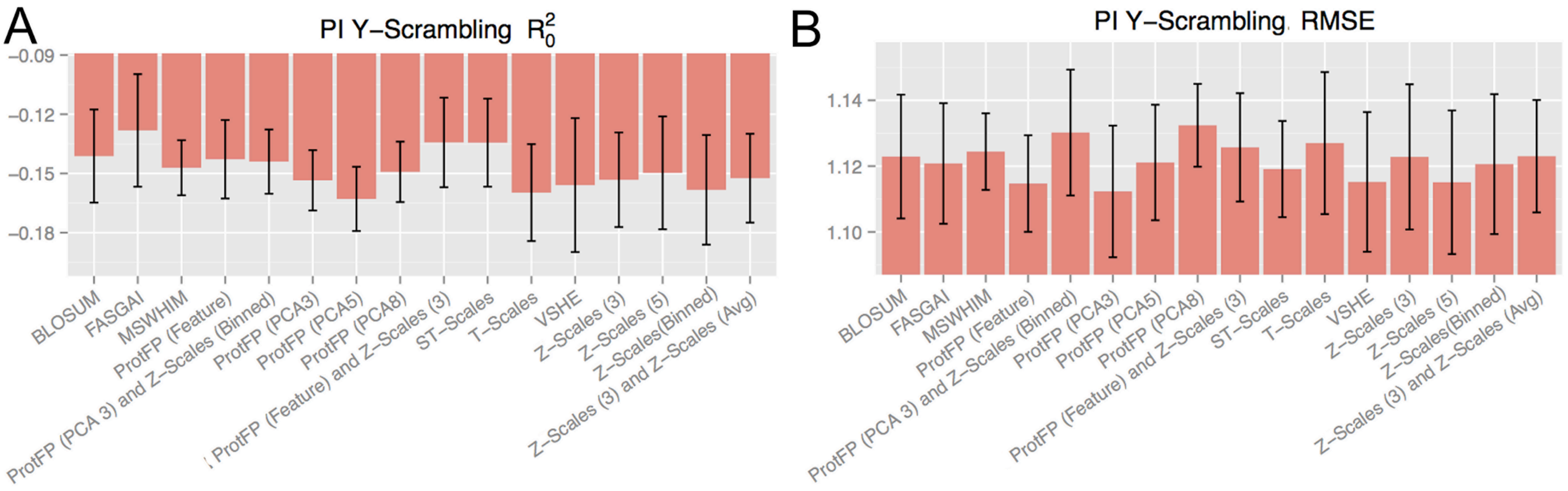


**Supporting figure S22.** PI 10-fold y-scrambling. Performance of the different descriptor sets on y-scrambled PI set.
